# Mario Coluzzi (1938–2012)

**DOI:** 10.1186/1475-2875-13-10

**Published:** 2014-01-22

**Authors:** Jeffrey R Powell, Nora J Besansky, Alessandra della Torre, Vincenzo Petrarca

**Affiliations:** 1 Department of Ecology and Evolutionary Biology, Yale University, New Haven, CT 06520, USA; 2 Eck Institute for Global Health & Department of Biological Sciences, University of Notre Dame, Notre Dame, IN 46556, USA; 3 Department of Public Health and Infectious Diseases, Università di Roma “La Sapienza”, Rome 00185, Italy; 4 Department of Biology and Biotechnology “Charles Darwin”, Università di Roma “La Sapienza”, Rome 00185 Italy

## 

Mario Coluzzi is well known to readers of this Journal as an outstanding medical entomologist, malariologist, epidemiologist, and perhaps less well known as an evolutionary biologist. He made important advances in a number of sub-disciplines and, equally importantly, inspired a large number of researchers who continue active research enterprises on the forefront of confronting tropical diseases. After a long struggle with Parkinson’s Disease, Mario Coluzzi died in Rome on October 20, 2012.

## Overarching contribution

When Mario Coluzzi was developing as a scientist in the 1950s and 60s, there was a radical and fundamental change occurring in population and evolutionary biology. Up until then evolution and systematics as well as medical entomology was dominated by “typological” thinking wherein practitioners identified entities such as species as having certain static properties that defined their essence. This typological view of species lumped together what in reality was a diverse set of genotypes that are spatially and temporally in flux. The new view was to recognize the actual diversity of the biological world down to the uniqueness of individuals. This was dubbed “populational” thinking. The consequences of this shift in viewing the living world has fundamental implications for all of biology. The two most influential leaders in this shift were Th. Dobzhansky (evolutionary biology and genetics) and E. Mayr (systematics). Given Coluzzi’s non-traditional, largely self-education, he read these and other kindred authors at a time when their views had yet to penetrate the practice of medical entomology and psitology.

As Coluzzi became more engaged in hands-on understanding of insect vectors, he saw how this new way of viewing diversity was crucial in understanding important medical problems. Indeed he recognized that the divide between what was considered academic versus applied or practical was downright damaging. He set out, and succeeded, to re-define the conception and practice medical entomology. The single theme that runs through all his work is the evolutionary *and* practical importance of vector analysis. Coluzzi consistently emphasized that vector analysis is important because vectors are neither the homogeneous nor static entities that much epidemiological modelling, experimental design, and even control programmes implicitly assume they are. He recognized (in fact, he demonstrated convincingly) that vector populations carry a tremendous amount of polymorphism, which implies genetic flexibility in adapting to heterogeneities in the environment, particularly those of anthropogenic origin. Differential responses to environmental heterogeneity lead to ecological adaptations and potentially reproductive isolation, which creates heterogeneity within and between vector populations. This is the *evolutionary* significance of vector analysis. More efficient utilization of the environment can also lead to increased total vector density and—by reducing intraspecific competition—to increased longevity, both of which result in heightened vectorial capacity. Differential responses within a vector species can also result in non-uniform exposure to control measures, lessening their efficacy. This is the *practical* significance of vector analysis. As defined by Coluzzi, the malaria challenge for medical entomologists is the problem of identifying and understanding the genetic diversity within vectors. He showed that the targets for control cannot always be recognized through rote application of alpha-taxonomy, based on rigid species definitions that do not account for the unique biology of these mosquitoes; they must be recognized through vector analysis (*sensu* Coluzzi).

Coluzzi’s first paper [1] reported DDT resistance in several anopheline species of Italy, including members of the *Anopheles maculipennis* complex. In that same year, (1958) George Davidson published two papers in *Nature*[2,3], the first of which reported a much higher level of dieldrin resistance in a colony of *Anopheles gambiae* from Nigeria (800-fold) than what had been measured in the field in Nigeria earlier that year (only 8-fold). The apparently lower resistance level in the field was hypothesized to result from the sampling of a mixed population of susceptible and resistant mosquitoes. One month later, the second paper reported the results of crossing studies designed to uncover the mechanism of dieldrin inheritance. The odd fact that *the male F1 hybrids were sterile* was interpreted initially as a side-effect of exposure to insecticide. These two *Nature* papers were followed eventually (not until 1962) by papers that finally revealed the fact that *A. gambiae* was actually a complex of at least 4 species. This momentous revelation was reported—not in *Nature*—but through World Health Organization (WHO) publications e.g., [4].

The slow and muted announcement of this discovery makes an interesting scientific and historical commentary, addressed by Coluzzi in his 1970 publication entitled “Sibling species and their importance in malariology” [5]. After citing older evidence, largely ignored, that vectors are heterogeneous, Coluzzi writes:

This slow progress … is certainly due, at least in part, to a lack of application of the new systematics concepts and techniques … [which] … have mostly remained on an academic plane. These important techniques are still not completely transferred from the hands of the geneticist to those of the medical entomologist. To stimulate this transfer is certainly an urgent need in malariology”.

It is rare that when looking at the history of a field, one can recognize a single event that has had fundamental lasting impact, or what historians of science call a pdigm shift. Coluzzi’s 1970 paper was the beginning of a true pdigm shift in medical entomology.

## Personal history

Mario Coluzzi was born November 30, 1938 in Perugia, Italy, to Alberto Coluzzi and Anna Wimmer. His father was a medical doctor who played a key role in eliminating malaria in the Monte Cassino Valley following World War II [6]. At the time, the Coluzzi family was living in the area in the villa “Casa delle Palme” in Monticelli. The villa was rented to the Italian Institute of Malariology at the symbolic cost of 1 Lira, and became the experimental station of the Institute. This villa played a key role in the early life of Mario. It was here that the young Mario started collecting insects using the cellar of the villa as an entomology museum and insectary. After accumulating numerous insects he wanted to arrange them in boxes; his father was willing to provide the boxes, but only for mosquitoes. Thus began a life-long focus on understanding Culicidae and their role in human history and health.

Mario married Adriana Sabatini in Rome on July 14, 1966. Adriana was herself a psitologist at the Istituto Superiore di Sanità, the Italian equivalent of the US National Institutes of Health. She continued work for many years including collaborating with Mario on key early publications. They had one daughter, Barbara, a Ph.D. in physics.

### Education

Mario Coluzzi’s formal education was, to say the least, unorthodox. He did graduate from the Liceo Scientifico Righi, one of the foremost science high schools in Rome at the time. That was the last formal degree he was awarded, although he was to receive two honorary degrees from Italian universities, a rare honor for a scientist. He believed in “learning by doing” and seemed almost proud of the fact he never graduated from University nor earned a higher degree. He did however study in both the Faculty of Medicine and Faculty of Biology at the University of Rome, La Sapienza. There he was most influenced by Ettore Biocca, the Director of the Institute of psitology. Throughout his schooling, he returned regularly to Monticelli to continue his studies of mosquitoes and informal education from his father.

### Career

From 1956 to 1966 Coluzzi worked at the Istituto di Malariologia “Ettore Marchiafava”. During this time he received a fellowship (1962–63) to work with the holder of the first Chair of Genetics in Italy, Giuseppe Montalenti. He also held an appointment from 1963–64 at the Istituto Superiore di Sanità.

In 1965, he returned to the University of Rome in Biocca’s Institute of psitology in the Faculty of Medicine where he was charged with organizing a laboratory of entomology that he headed from that time forward. He began traveling and spent time working in England hosted by Peter Mattingly and George Davidson, and in France hosted by Jean Rioux. In 1968, Mario attended the WHO course on vector genetics organized at the University of Notre Dame by George B. Craig, Jr.

In 1975, Mario took a faculty position at the University of Camerino, some 200 kilometers northeast of Rome. He attained the rank of Full Professor in 1982. While teaching and attending to administrative duties in Camerino, he maintained an active research program at the University of Rome. He returned full time to La Sapienza in 1982. Upon retirement of his old mentor, Ettore Biocca, he became Director of the Institute of psitology in 1987. He remained in this position until retiring in 2010.

### Honors and society memberships

•Member of the Società Italiana di Entomologia (1956–1998) and member of the editorial board of its Journal.

•Member of the Società Italiana di pssitologia since 1952; Executive Committee since 1982, Vice-President (1984–1988), President (1988–2000).

•Member of the Royal Society of Tropical Medicine and Hygiene since 1966.

•'Chalmers Medal’ (1982) of the Royal Society of Tropical Medicine and Hygiene.

•Member of the Accademia Nazionale di Entomologia since 1986.

•Member of the Accademia Medica di Roma since 1989.

•Winner of the 'Premio Feltrinelli per la Medicina’ (1989) from the Accademia Nazionale dei Lincei.

•Member of the Accademia Nazionale dei Lincei since 1995.

•Honorary degree in Medicina e Chirurgia (1998) from the Università di Tor Vergata, Rome, Italy.

•'Ronald Ross Medal’ (1998) from the London School of Hygiene and Tropical Medicine.

•'Mary Kinsley Award’ (1998) from the Liverpool School of Tropical Medicine.

•Honorary degree in Natural Sciences (1999) from the Università di Camerino.

•'Prix Emile Brumpt’ (2003) from the Societé Française de psitologie.

•Harry Hoogstraal Medal for Outstanding Achievement in Medical Entomology (2006) from the American Society of Tropical Medicine and Hygiene.

•Life-time Achievement Award (2007) from the BioMalPar EU Network of Excellence.

•Medaglia Montalenti, Università di Roma La Sapienza (2008).

## Africa

There is no need to remind readers of this Journal that sub-Saharan Africa is the most important region of the world with regard to human malaria, and it was here that Mario Coluzzi made his most important and lasting contributions.

**Figure 1:**
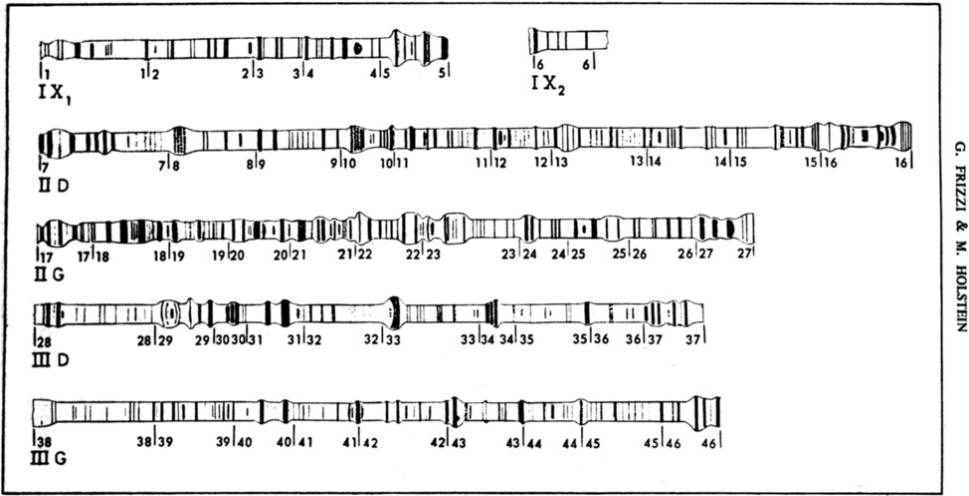
**Low resolution larval polytene chromosome map of *Anopheles gambiae s.l., *** **from [**[8]**].**

**Figure 2:**
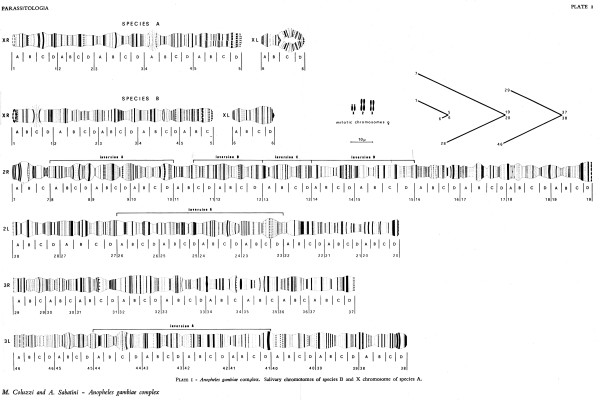
**Higher resolution map of *A. gambiae *** **(sp. A) and *A. arabiensis *** **(sp. B) larval polytene chromosomes, from [**[7]**].**

Beginning in 1966, Coluzzi and his wife Adriana Sabatini began to work with *A. gambiae* salivary chromosomes. A real break-through came in October of 1967 with the first publication by Coluzzi and Sabatini [7] of what was to be a series of papers that presented detailed cytogenetic maps based on the banding patterns of polytene chromosomes found in the larval salivary glands of members of the *A. gambiae* complex. Previous cytogenetic work on these species [8] had provided comptively low resolution maps (Figure 1). Figure 2 shows the much higher resolution maps that arose from work by Coluzzi and Sabatini on what was called “species A” and “species B” (later named *A. gambiae* and *Anopheles arabiensis*) [7].

**Figure 3:**
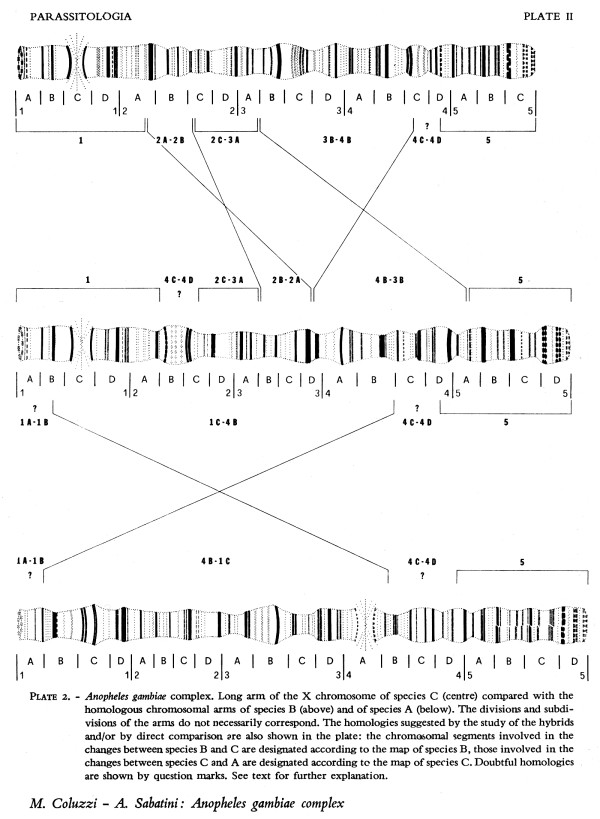
**Comparison of larval polytene X chromosome banding pattern homologies between *A. quadriannulatus *** **(sp. C), *A. gambiae *** **(sp. A) and *A. arabiensis *** **(sp. B), from [**[9]**].**

In comparing the X chromosome maps among the freshwater species (species A, B and C; Figure 3), Coluzzi realized that they differed by fixed pcentric inversions [9]. These, together with fixed autosomal differences septing the freshwater and saltwater species [10], provided the first diagnostic tool (a cytotaxonomic one) that could be used to study field populations. Until this time, the only way to distinguish members of the complex was by laborious crosses that could only be performed using laboratory colonies; no reliable morphological differences were ever found at any developmental stage despite a concerted effort by Coluzzi [11] and others. An even more powerful advance came with the discovery by Coluzzi [12] of very favourable polytene chromosomes in the ovarian nurse cells of adult females. Previously, reliance on larval salivary gland chromosomes during an epidemiological investigation compelled malariologists to rear the progeny of wild-caught gravid adult females, a process that cost up to ten days before a positive identification could be made. Discovery of ovarian polytene chromosomes meant that cytotaxonomy and measurement of epidemiologically relevant pmeters could be performed directly on wild-caught females.

In addition to their practical utility, these data were used to derive phylogenetic relationships among the five species [10]. The phylogenetic hypothesis suggested two very surprising things. First, ecologically similar species pairs were not necessarily the most closely related evolutionarily, implying that similar lifestyles arose secondarily, from independent speciation processes. Thus, *Anopheles merus* and *Anopheles melas*, both saline-tolerant species that breed in the brackish coastal margins on opposite sides of the continent, arose independently. Similarly, *A. arabiensis* and *A. gambiae*, both major vectors with widespread and largely overlapping distributions, also arose independently. Second, polymorphic chromosomal inversions common to *A. arabiensis* and *A. gambiae* likely were shared due to residual gene flow and genetic introgression subsequent to speciation. Today there is growing acceptance that under certain conditions, animal species can form and persist in the absence of geographic barriers, even in the face of some ongoing hybridization and introgression. In 1969, few zoologists except Guy Bush (who championed sympatric speciation of the phytophagous insect *Rhagoletis pomonella* in his 1966 PhD thesis) would have been open to this suggestion. Common wisdom dictated that animal speciation required physical seption between populations, and any gene flow would either reverse the speciation process or produce unfit hybrids (evolutionary dead-ends). On the contrary, Coluzzi argued that hybridization and introgression had a constructive role in the origin of the species known today as *A. gambiae*[13]. He envisioned that an anthropophilic *A. gambiae* arose in the Central African rainforest, where human agricultural activity would have broken the forest canopy and created the sunlit pools required for *A. gambiae* breeding. Emergence of *A. gambiae* from the humid rainforest into dry savannas would have been made possible only by hybridization with the savanna-adapted *A. arabiensis* and the consequent introgression of chromosomal inversions 2Rb and 2La that are associated with aridity tolerance [13] (see below). Coluzzi’s insights are even more impressive in light of the fact that his main tool—aside from polytene chromosome analysis—was his deep “feeling for the organism”; only many years later when DNA-based evidence became available were his views vindicated.

**Figure 4:**
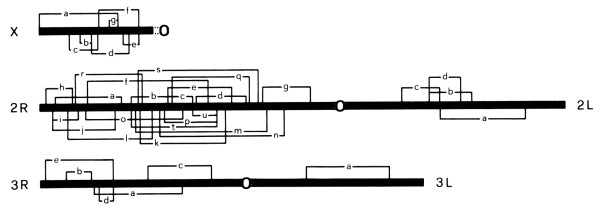
**Schematic representation of polytene chromosome complement with both fixed and polymorphic inversions observed in six species of the *A. gambiae *** **complex, from [**[14]**].**

Further advancements ensued from his species-level polytene chromosome analysis. Figure 4, from his classic 1979 paper [14], shows the distribution of the more common inversions observed in the species complex. A non-random pattern is clearly evident. If inversions were random and selectively neutral, their expected distribution would be proportional to chromosome length. This was not the case. Coluzzi noted that fixed inversion differences between species were disproportionately found on the X chromosome; it carries five of 10 fixed inversions. Furthermore, he realized that species differed either by fixed inversions on the X or the autosomes but not both. The two allopatric saline-tolerant species differed from the freshwater breeders in their autosomal banding patterns. On the other hand, the strictly sympatric freshwater species differed by X chromosome banding patterns. This led Coluzzi to hypothesize that the X is the source of genes involved in reproductive isolating barriers, a hypothesis called “the large X-effect” by *Drosophila* workers, subsequently called a “rule” of speciation [15]. Autosomes, on the other hand, were posited by Coluzzi to be the primary source of genes involved in ecological adaptations involving the larval habitat.

Not only fixed inversions, but also polymorphic inversions were non-randomly distributed across the genome. These are overrepresented on the right arm of chromosome 2 in the *A. gambiae* complex (Figure 4) and in *A. gambiae sensu stricto*[16]. Moreover, their breakpoints are non-randomly distributed, and in some cases apparently coincident. Coluzzi proposed that the particular region covered by essentially all 2R rearrangements differentiating species and forms of *A. gambiae*, contains genes controlling optimal larval habitat or oviposition preference, as this seems to be the salient characteristic by which these taxa differ [17]. This intriguing hypothesis can now be tested, given whole genome reference sequences newly available for six members of the *A. gambiae* complex [18-20].

Coluzzi also noted a striking difference in the abundance of polymorphic inversions between members of the *A. gambiae* complex. At one extreme, *A. merus* and *Anopheles amharicus* (formerly *Anopheles quadriannulatus* B; [21]) lack inversion polymorphisms altogether, and *A. quadriannulatus* is relatively depauperate of polymorphism. At the other extreme are *A. arabiensis* and *A. gambiae*, the most geographically widespread and ecologically dominant members of the complex across the heterogeneous landscapes of tropical Africa. To Coluzzi, this was no accident, but rather a consequence of abundant inversion polymorphism, which allows “*greater ecological flexibility and more efficient exploitation of different niches … through the capture and stabilization within inversions of blocks of co-adapted genes*.” [14].

**Figure 5:**
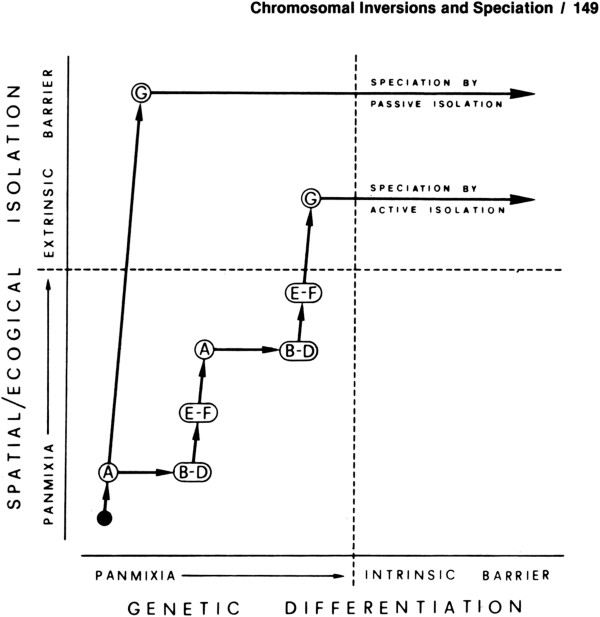
**Coluzzi’s model of chromosomal speciation in anopheline mosquitoes, from [**[22]**].**

In 1982, Coluzzi made a seminal contribution to speciation theory when he first put forth a verbal model of chromosomal speciation [22] (Figure 5). The basis of this model is the idea that marginal populations (those at the fringe of acceptable habitat, “A” in Figure 5) become locally adapted. If an inversion subsequently arises that captures those locally adapted alleles (B-D in Figure 5), it can preserve them from recombination into the genetic background of core populations during periods when marginal and core populations come into contact, facilitating an extension of the locally adapted marginal population into even more “hostile” or extreme habitat (E-F in Figure 5). Ultimately, an extrinsic barrier is achieved between the new isolate and the parental population. In the Coluzzi model [22], adaptive genetic changes precede and facilitate extrinsic (ecological) isolation, while in classic allopatric speciation, physical seption imposed by a major geographic barrier (e.g., ocean or mountain range) precedes genomic differentiation. More recently, chromosomal speciation theories based on sunflowers, *Drosophila*, and even humans have been published to great fanfare [23-26]. Yet few are aware of Coluzzi’s model, fully twenty years ahead of its time.

In 1982, Coluzzi stopped short of actually calling his model a form of sympatric or ecological speciation, but he continued to hold what was then considered to be a progressive view on the matter of speciation [27]. In fact, there is evidence that he held this progressive view much earlier. In an exchange between Garrett-Jones and Coluzzi recorded in 1970, Garret-Jones remarked: “*I don’t fully understand why, if you find a fully fertile cross between two mosquitoes which are morphologically indistinguishable, that does not prove that they are conspecific*.” Coluzzi responded, “*Sterility barriers are, I would say, the most important mechanisms for reproductive isolation but not the only and not necessarily the first to develop. We may have reproductive isolation between two populations without sterility barriers*.” [5], p.76. This anticipates and foreshadows elucidation of the chromosomal forms of *A. gambiae* by Coluzzi and colleagues [28], and encapsulates the protracted controversy over them. It also underscores what Coluzzi meant by his oft-repeated phrase “vector analysis” [29] and why typological thinking can be terribly misleading to malariologists.

**Figure 6:**
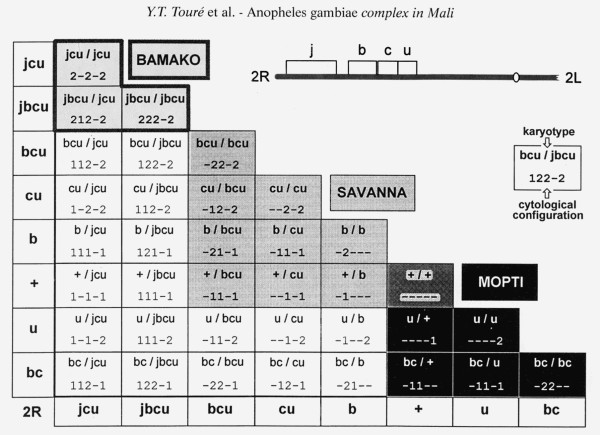
**Karyotypes and polytene chromosome configurations expected if the eight chromosome 2R rearrangements (inverted and standard orientations of 2Rj, b, c and u) combined at random.** Shading represents three chromosomal forms of *A. gambiae* observed in Banambani, Mali. From [30].

**Figure 7:**
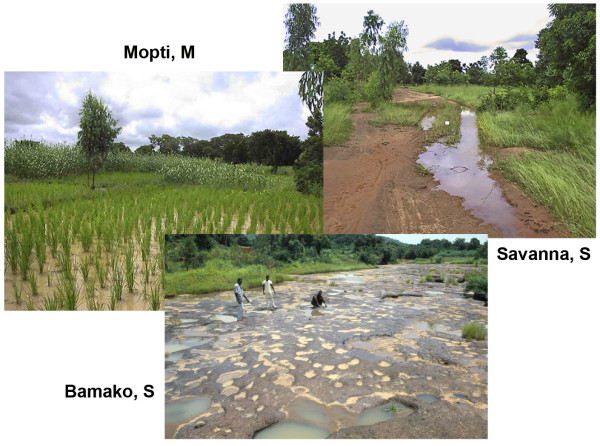
**Typical larval habitats of the chromosomal/molecular forms of *A. gambiae *in West Africa.** Mopti chromosomal form (M molecular form, now A. coluzzii [21]) breeds in rice fields (Photo by C. Costantini). Savanna chromosomal form (S molecular form, now *A. gambiae s.s*.) breeds in rain-dependent temporary pools and puddles (Photo by C. Costantini). Bamako chromosomal form (S molecular form) breeds in laterite rock pools beside the Niger River (Photo by M. Fodde and M. Coluzzi).

Yeya Touré, Joan Bryan and others working with Coluzzi discovered non-random patterns of inversion polymorphism within *A. gambiae s.s*. from West Africa, consistent with the earliest stages of speciation. Figure 6 shows the situation in Mali [30], where not all of the 36 possible inversion combinations involving chromosome 2R are found at the frequencies expected given a single randomly mating population. Departures from Hardy-Weinberg equilibrium, owing to marked heterozygote deficiencies, were resolved if the data were assumed to come from three septe assortatively mating populations referred to as “chromosomal forms” and designated Bamako, Savanna and Mopti. These forms were distinguished by characteristic combinations of shared inversions, and were associated with different larval ecologies—temporary rain-filled pools and puddles away from major rivers (Savanna); laterite rock pools beside the Niger River in Mali and Guinea (Bamako); or stable sites associated with irrigated agriculture such as rice fields (Mopti) (Figure 7). Eventually, sequence differences in ribosomal DNA genes were found by collaborators of Coluzzi to distinguish Mopti and Savanna populations from Mali and Burkina Faso [31]. However, karyotype-based definitions were later shown to be incongruent with rDNA-based divergence, leading to the definition of “M- and S- molecular forms” [32], and raising controversies regarding the precise role of 2R pcentric inversions in the speciation process within *A. gambiae s.s.*[33].

Based on genome-wide patterns of sequence divergence and bionomic evidence that M and S are cohesive and exclusive taxonomic groups, the name *Anopheles coluzzii* Coetzee & Wilkerson sp.n. was recently assigned to the M-form [21], and references therein, to recognize the seminal contribution of Mario Coluzzi in highlighting the complexity within *A. gambiae sensu lato* and its considerable implications in malaria epidemiology in sub-Saharan Africa.

**Figure 8:**
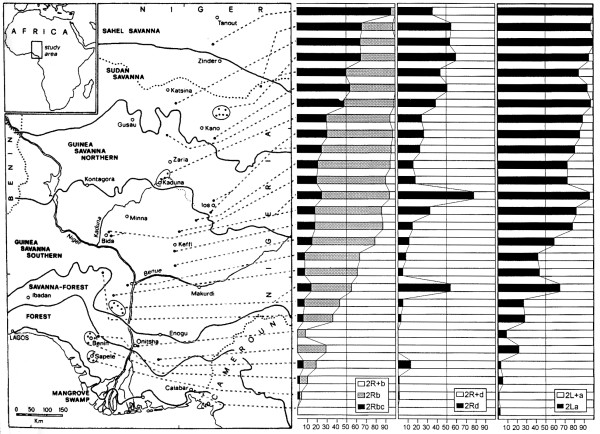
**Clinal variation in chromosome 2 inversion frequencies along an environmental gradient of increasing aridity, from coastal Nigeria in the south to the sahel savanna of Niger in the north.** Data from [14], as depicted by [13].

In addition to their role in speciation, chromosomal inversions are also important means by which adaptive variation is maintained within species. Coluzzi and colleagues showed clear and highly significant correlations between inversion frequency and degree of aridity. Shown in Figure 8 are frequencies of 2^nd^ chromosome inversions from ~24,000 *A. gambiae* sampled from 194 localities along a north–south transect from coastal Nigeria through Niger in West Africa [14]. With increasing aridity from the humid rainforest to the dry savanna, chromosome 2 inversion frequencies shift from near absence to fixation. Similar climate-related changes in inversion frequencies are also manifest locally, and are correlated not only with shifts in rainfall between rainy and dry seasons, but even with shifts in microclimate that influence mosquito resting behavior at the scale of a village. As the saturation deficit is higher indoors at night than outdoors, inversion frequencies are higher among mosquitoes resting indoors than those captured outdoors. The practical importance of this inversion-associated influence on indoor resting behavior was a lesson derived by Coluzzi from the Garki Project [34], as it implies a non-random exposure by malaria-transmitting mosquitoes to vector control methods such as bed nets and indoor residual insecticide. The stability of these trends across years and in different parts of Africa suggests that the distribution of inversion-bearing mosquitoes in the environment is a response to spatially varying selection pressures mediated by the inversions themselves.

By the late 1980s, public appreciation of the importance of vector-borne diseases was experiencing a renaissance. Great interest was developing that stimulated increased funding by prominent foundations and the recruitment into the field of prominent molecular biologists and geneticists previously working in basic research on other organisms [35]. Coluzzi’s previous 20 years of work on *A. gambiae* in Africa made this system a forefront of the renaissance. It is no coincidence that, in 2002, *An. gambiae* was the first mosquito (indeed, only the second insect after *Drosophila melanogaster*) to have its complete genome sequenced and assembled [18], an effort greatly facilitated by the polytene chromosome map that Coluzzi and colleagues painstakingly developed [36]. Mario Coluzzi has been rightly dubbed the “Father” of modern medical entomology in Africa.

## Coluzzi in the field

**Figure 9:**
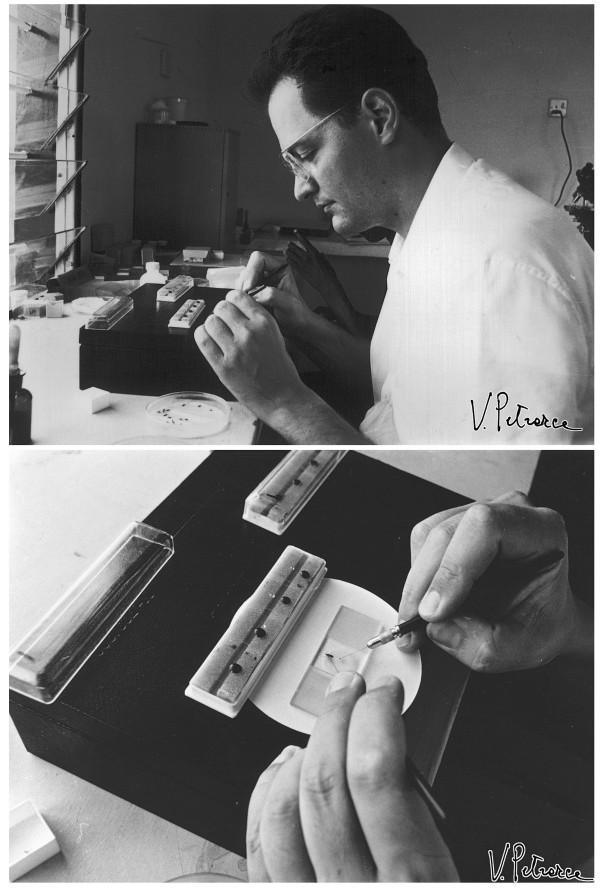
**Coluzzi dissecting ovaries of *Anopheles gambiae s.l*. at a laboratory of the Garki Project, Nigeria, August 1971.** (Photo by V. Petrarca).

**Figure 10:**
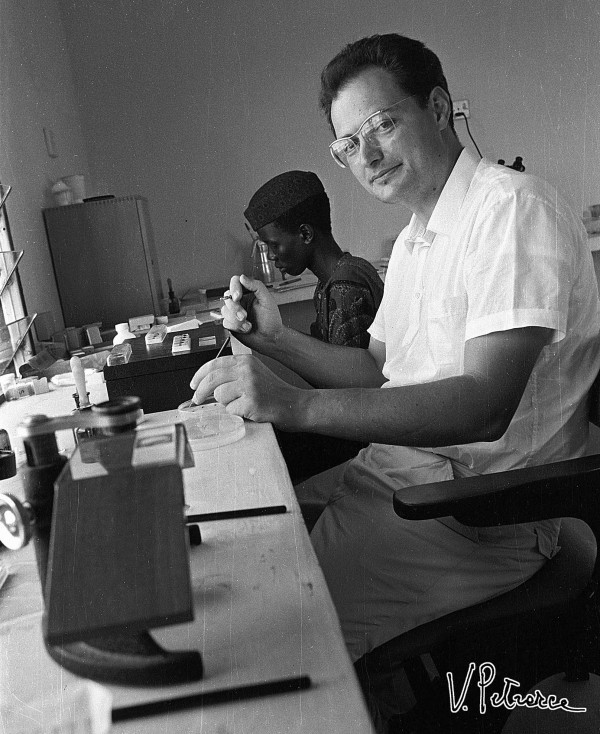
**Coluzzi dissecting mosquitoes at a laboratory of the Garki Project, assisted by a local young man in Garki, Nigeria, August 1971.** (Photo by V. Petrarca).

Mario Coluzzi was surely favoured for fieldwork by his physique and by having been neither a big eater, drinker, or smoker. Mario’s sturdiness and tenacity allowed him to pursue his goals without distractions and without showing obvious signs of fatigue, even after long working hours in warm climates. Moreover, in the 1970s Coluzzi’s hands were so steady and his eyesight so sharp (even if aided by spectacles) that he was able to dissect mosquito ovaries to obtain polytene chromosomes from nurse cell nuclei without the aid of a microscope (Figure 9), even the simplest one (foreground of Figure 10). Humorously, after having seen Coluzzi at work on a bench of the Garki Project’s laboratory, a WHO British colleague began to call him “immersion-oil-eyes Mario”.

**Figure 11:**
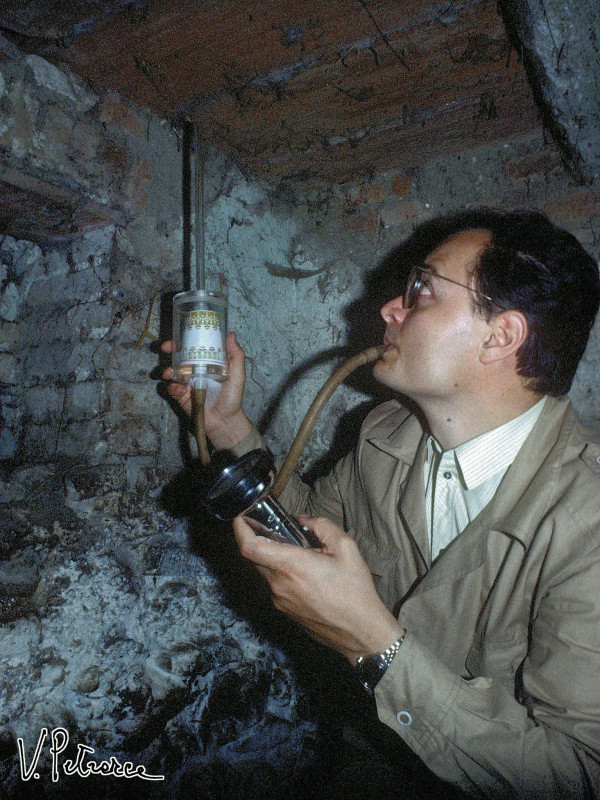
**Coluzzi collecting adult *Anopheles maculipennis *in a pigsty at Orte (Viterbo, Italy), summer 1978.** (Photo by V. Petrarca).

**Figure 12:**
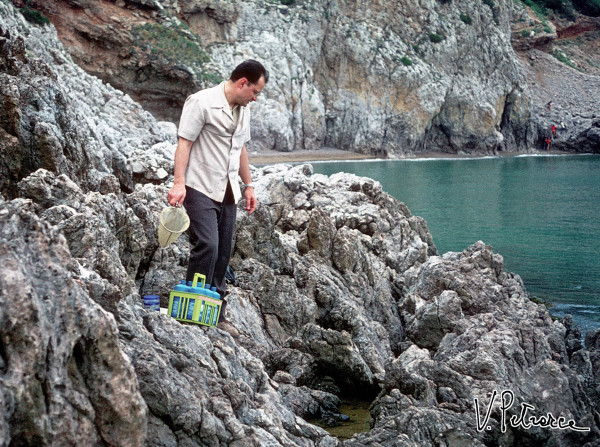
**Coluzzi sampling *Aedes mariae *larvae in coastal rock pools at Torre Capovento (Sperlonga, Latina, Italy), June 1975.** (Photo by V. Petrarca).

**Figure 13:**
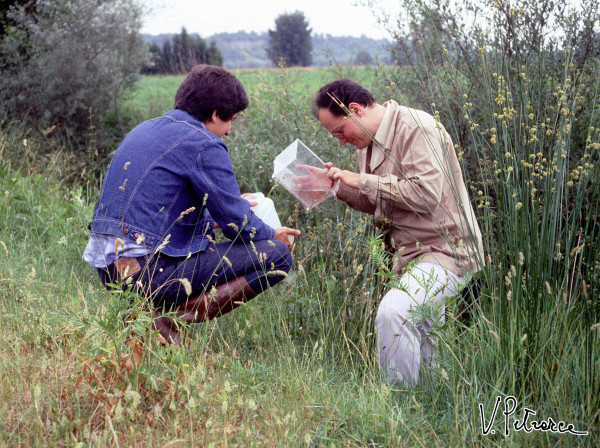
**Looking for larvae of the *Anopheles maculipennis *complex in the countryside of Orte (Viterbo, Italy), summer 1978.** (Photo by V. Petrarca).

Mario’s knowledge of the ecosystems of Palearctic mosquito species was vast and deep. With the same enthusiasm, proficiency, and efficiency he could successfully: look for very aggressive *Aedes (Ochlerotatus) cataphylla* at the edge of a glacier; slip into stinking sewerage chasing *Culex pipiens*; face fierce pigs to recover a few *A. maculipennis* in tiny pigsties (Figure 11); risk his neck clambering up steep intertidal rocks to find some *Aedes mariae* (Figure 12); get dirty from head to toe with the black and smelly water from tree holes in search of *Ochlerotatus geniculatus* and *Anopheles plumbeus* larvae; get lost and muddy in a swamp running after *Anopheles labranchiae* (Figure 13), etc. But his lifelong love was for Africa and the *A. gambiae* complex.

Typically, the first thing he did when arriving in a new village in Africa, was to have a meeting with the intrigued chief of the village, explaining to him in a very simple and clear way what his intentions were and what he intended to achieve, requesting the chief at the same time to apologize to the inhabitants of the houses that were to be visited for any possible inconveniences. However, usually the villagers were far from being unhappy, especially during and after pyrethrum spray collections, because they would be freed for several days from being tormented and bitten by hundreds of mosquitoes every night or being annoyed by countless other insects, centipedes, spiders, and scorpions.

**Figure 14:**
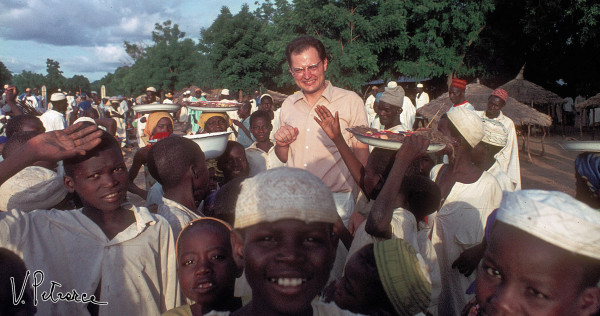
**Coluzzi in the marketplace at Garki village, Nigeria, August 1971.** (Photo by V. Petrarca).

**Figure 15:**
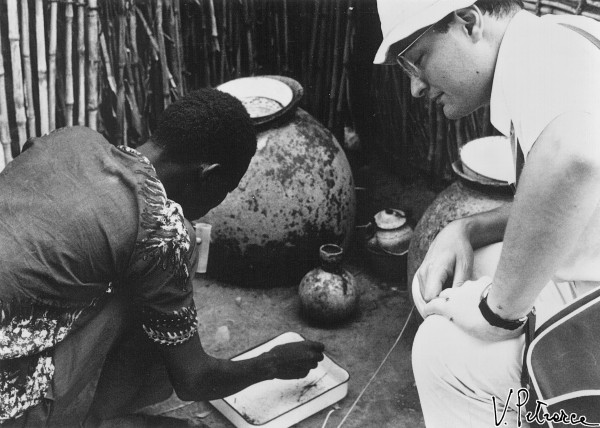
**Coluzzi training a local assistant in collecting *Anopheles gambiae s.l*. larvae at Matsari village (Garki, Nigeria), August 1971.** (Photo by V. Petrarca).

His second task was to identify among children and teenagers (inevitably surrounding in droves the alien visitors, sometimes the first Caucasians who the youngsters had seen in their lives) those who could be of help in identifying the best places to search and capture mosquitoes (Figure 14). Mario’s experience was that very often among teenagers were those with highly developed powers of observation that could have been of great support, and he was almost always correct. Often it was possible to involve highly trained technicians for both the field and the laboratory activities (e.g., the case of the Garki Project in Nigeria), but whenever this was not possible, Mario spent much time training the people that would help him, involving young villagers too (Figures 10, 15). This was not just an investment of time aimed at more efficient collecting; Coluzzi had an natural propensity for teaching, improved in the course of time, supported by an incompble ability to fascinate the audience, helped by knowing well both English and French.

The eventual consequence of these preliminary efforts was that sometimes the entire population of village youth were organized into a single enthusiastic body chasing and capturing mosquitoes, under the amused and sometimes perplexed eyes of the chief and elders of the village, with whom, during the breaks in work, Mario chatted about everyday life, cattle breeding, poisonous snake threat, rainfall delay, millet cultivation, etc.

Native villagers were particularly struck by Coluzzi’s ability to “think like a mosquito”, a result of innate powers of observation, deep understanding of nature, and an indefatigable dedication to his purposes. Many colleagues also picked up on this, jokingly and admiringly referring to his ability to think like a mosquito. One of Coluzzi’s most valuable traits was the will and the ability to support and promote young students and researchers, particularly from Africa, who attracted his attention for their motivation, potential, skill and intelligence. He stimulated their autonomy (but always with great respect for local cultural authorities, avoiding frictions of any kind), enhanced their powers of observation, invited them to the Rome laboratory, and supported them with training grants, aimed at enhancing and strengthening their cultural preption. The result is that today many of them are working in international health agencies, in Africa and around the world.

A final, somewhat frightening, memory: Mario drove to sampling sites at a very, very high speed, chatting directly with the passenger (about mosquitoes, of course) not looking at the road: the passenger was always scared to death by that habit. Anyone who rode with Coluzzi when visiting Rome had similar experiences.

## Human research

Given Coluzzi’s conviction of the central importance of genetic variation in understanding the malaria problem in sub-Saharan Africa, decades of work in the field made it abundantly clear that it is not only genetic variation of the mosquito that is important, but also that genetic variation in the human host is of equal or greater importance. Coluzzi was fortunate in identifying a highly competent young Italian malaria epidemiologist with considerable knowledge of human genetics, David Modiano, with whom he made significant contributions in this realm. (No doubt if he had been able to enlist a protozoologist, he would have investigated the role of genetic variation in the malaria psite, as well).

One of their first studies was in Ouagadougou, Burkina Faso where three distinct ethnic groups were living together. They were all uniformly exposed to high levels of infection with *Plasmodium falciparum*, yet their immune responses were very different. All human genetic variants known to be involved in resistance to malaria were ruled out as being responsible. Thus they revealed a high level of previously unknown human genetic variation with regard to susceptibility and response to infection with the malaria psite [37].

Sickle-cell anemia caused by the HbS allele of hemoglobin has long been known as a classic human variant associated with resistance to malaria in sub-Saharan Africa. Another hemoglobin variant, HbC, was studied by Modiano et al. [38] and was shown to confer very similar resistance to infection with *P. falciparum* as HbS, but without the highly deleterious effects in homozygotes. Thus it was predicted that HbC should eventually replace HbS if West Africa continues to experience *P. falciparum* epidemics.

A second line of human-related work consumed much of Coluzzi’s later years. This concerned the potential role of blood-sucking insects in transmission of HHV8 virus. Coluzzi and colleagues put forward the hypothesis that viruses transmitted from parents to children via licking wounds or irritated areas with saliva (a common practice in Africa) would be enhanced by blood-feeding arthropods [39-41]. Not only would the irritation caused by bites increase licking, but also the arthropod saliva reduces the immune response at the site of bites and the recruitment of inflammatory cells. Coluzzi called this the “promoter-arthropod” hypothesis to highlight its distinction from the direct transmission of pathogens by arthropods.

Finally, Coluzzi’s last work on humans concerned the influence of a mutation in the human CYPC8*2 gene that leads carriers to metabolize the anti-malarial drugs amodiaquine and chloroquine more slowly than normal [42]. The consequence is two fold: increased drug-associated side effects and increased selection for resistance to these drugs by the *P. falciparum* psites. Coluzzi’s final publication is on this subject and appears in this Journal [43].

## Coluzzi in Italy

In addition to the tremendous amount of work accomplished in Africa, it is remarkable that Mario Coluzzi almost continuously maintained a number of other projects primarily in Italy.

Early studies (1956–61) concerned the susceptibility of Italian populations of *Anopheles* to DDT [1,44-46]. It was observed that virtually no change in susceptibility evolved after nine rounds/year of indoor spraying over several years; in the laboratory this level of selection led to considerable resistance. Coluzzi and colleagues concluded that selection in the field had occurred in response to the irritant effect of DDT, such that mosquitoes simply left the indoor environment rather than evolve physiological resistance. In fact, this change in behaviour in response to DDT’s irritant effect was sufficient to disrupt malaria transmission.

Malaria was a problem in Italy and elsewhere in Europe when Coluzzi was born, and he studied various European anophelines capable of malaria transmission, primarily the *An. maculipenis* complex [47]. Bietolini et al. [48] is Coluzzi’s final paper on the *maculipennis* complex. This paper is a compilation of long-term records of distributions coupled with climate change models predicting distributions in ~2050.

Coluzzi also investigated morphological traits that could be relevant for mosquito fitness and capacity for pathogen transmission. For instance, in comparing cibarial armatures in different mosquito genera/species he showed that different morphologies determine levels of haemolysis after blood meals ranging from 5 to 50%, thus supporting the hypothesis of a mechanical action of cibarial armatures on erythrocytes and eventually blood metabolism [49].

Coluzzi carried out studies of canine *Dirofilaria* in Italy over several years. Among other things, he demonstrated for the first time transmission by tabanid flies in addition to mosquitoes [50], and with colleagues developed PCR diagnostic procedures distinguishing species of psite worms [51].

In the early 1970s, Coluzzi began a fruitful collaboration with Luciano Bullini who was using the then recently developed allozyme technology to study population genetics and mosquito behavior. This work focused primarily on the Italian coastal mosquito, *Aedes mariae* (Figure 9), and resulted in some of the first publications demonstrating the use of allozymes to detect mating barriers between closely related taxa [52-54]. This clearly demonstrated Coluzzi’s interest in speciation problems that were later developed so successfully in his African work discussed above.

A complete bibliography of Mario Coluzzi's publications is available as Additional File 1.

## Supplementary Material

Additional file 1 Mario Coluzzi - Publications 1956 - 2012. Click here for file
