# Supplementary material for: Mario Coluzzi (1938–2012)
Source: Malar J. 2014 Jan 22;13:10. doi: 10.1186/1475-2875-13-10 (PMC3914365; doi:10.1186/1475-2875-13-10)
Supplement: Additional file 1 — Mario Coluzzi - Publications 1956 - 2012. [file 1475-2875-13-10-S1.doc]

**Mario coluzzi**

**Publications 1956 - 2012**

1. RAFFAELE G., COLUZZI M. (1956). Esperienze sulla resistenza al DDT delle specie di anofeli di varie regioni d'Italia. Rivista di Malariologia, 35: 177-198.
2. RAFFAELE G., COLUZZI M. (1957). Ricerche sul problema della resistenza degli anofeli agli insetticidi. Nota I. Alcune osservazioni sulle modalità di attuazione del metodo di Busvine e Nash. Rivista di Malariologia, 36: 157-166.
3. RAFFAELE G., COLUZZI M. (1957). Ricerche sul problema della resistenza degli anofeli agli insetticidi. Nota II. Su alcune variazioni della sensibilità degli anofeli al DDT. Rivista di Malariologia, 36: 167-176.
4. RAFFAELE G., COLUZZI M. (1957). Ricerche sul problema della resistenza degli anofeli agli insetticidi. Nota III. Indagini sulla sensibilità al DDT degli anofeli di varie regioni d'Italia dopo 10 anni di trattamento con insetticidi. Rivista di Malariologia, 36: 177‑202.
5. MAFFI M., COLUZZI M. (1958). *Anopheles* (*Neocellia*) *salbaii* n. sp. Morfologia ed ambiente. Rivista di Malariologia, 37: 45-55.
6. COLUZZI M. (1958). Descrizione di una nuova specie di *Anopheles* rinvenuta in Somalia. Rivista di Malariologia, 37: 69-71.
7. COLUZZI M., RUGGIERO C. (1958). Indagine sulla sensibilità al DDT di alcune specie di anofeli della Somalia. Rivista di Malariologia, 37: 77-83.
8. COLUZZI M. (1958). Nota pratica. Un metodo "standard" per ottenere facilmente ovodeposizioni. Trasporto e montaggio degli anofeli. Rivista di Malariologia, 37: 85-89.
9. RAFFAELE G., COLUZZI M. (1958). Ricerche sul problema della resistenza degli anofeli agli insetticidi. Nota IV. Ulteriori considerazioni sulla sensibilità degli anofeli italiani al DDT in relazione ai risultati ottenuti con diversi metodi. Rivista di Malariologia, 37: 193-198.
10. COLUZZI M. (1958). Dati sperimentali sulla irritabilità di diverse specie di anofeli a contatto con pareti trattate con DDT. Rivista di Malariologia, 37: 199-228.
11. MAFFI M., COLUZZI M. (1960). *Anopheles macmahoni* Evans, nella regione della Migiurtinia, Somalia. Rivista di Malariologia, 39: 119-129.
12. COLUZZI M. (1960). Alcuni dati morfologici e biologici sulle forme italiane di *Anopheles claviger* Meigen. Rivista di Malariologia, 39: 221-235.
13. COLUZZI M. (1960). Presenza in Italia continentale di *Theobaldia morsitans* Theobald e di *T. subochrea* Edwards (Diptera, Culicidae). Bollettino della Società Entomologica Italiana, 90: 152-155.
14. COLUZZI M. (1961). Nota sulla biologia di *Uranotaenia* (*Uranotaenia*) *unguiculata* Edwards. Primo reperto della specie in Sicilia (Diptera, Culicidae). Bollettino della Società Entomologica Italiana, 91: 21-24.
15. COLUZZI M. (1961). Sulla presenza di *Culex* (*Culex*) *theileri* Theobald in Italia centrale, meridionale ed in Sicilia (Diptera, Culicidae). Bollettino della Società Entomologica Italiana, 91: 55-57.
16. COLUZZI M. (1961). Sulla presenza in Italia di *Aedes* (*Finlaya*) *echinus* e di *Aedes* (*Stegomyia*) *vittatus* (Diptera, Culicidae). Bollettino della Società Entomologica Italiana, 91: 77-79.
17. COLUZZI A., COLUZZI M. (1961). Sull'irritabilità degli *Anopheles* al DDT in rapporto all'eradicazione della malaria. Rivista di Malariologia, 40: 35-40.
18. RAFFAELE G., COLUZZI M. (1961). Su *Anopheles* (*Myzomyia*) *hispaniola* Theobald, 1903 e *Anopheles* (*Myzomyia*) *cinereus* Theobald, 1901. Rivista di Malariologia, 40: 247‑251.
19. COLUZZI M. (1961). Su alcuni esemplari etiopici di *Anopheles machmahoni* Evans, 1936. Rivista di Malariologia, 40: 253-256.
20. COLUZZI M. (1962). Su alcuni Culicini poco noti o non segnalati in Italia (Diptera, Culicidae). Parassitologia, 4: 13-22.
21. RIVOSECCHI L., COLUZZI M. (1962). Tre simulidi (*Simulium aureum* Fries (s.l.), *Simulium erythrocephalum* de Geer, *Simulium reptans* L.) che in Italia pungono l'uomo. Parassitologia, 4: 181-190
22. COLUZZI M., RIOUX J.A. (1962). Primo reperto in Italia di larve di *Anopheles* parassitate da funghi del genere *Coelomomyces* Keilin. Descrizione di *Coelomomyces raffaelei* n. sp. (Blastocladiales, Coelomomycetaceae). Rivista di Malariologia, 41: 29-37.
23. VALENTI M., COLUZZI M. (1962). Anofelismo residuo nel comune di Roma a dieci anni dalla scomparsa dell'endemia malarica. Rivista di Malariologia, 41: 69-73.
24. COLUZZI M. (1962). Sulla modalità di applicazione della tecnica di McDaniel e Horsfall all'accopppiamento artificiale delle zanzare. Rivista di Malariologia, 41: 75-78.
25. COLUZZI M., CONTINI C. (1962). The larva and pupa of *Mansonia* (*Coquillettidia*) *buxtoni* (Edwards), 1923, (Diptera, Culicidae). Bulletin of Entomological Research, 53: 215‑218.
26. VALENTI M., COLUZZI M. (1962). Contributo alla conoscenza dei culicidi della città e del comune di Roma. Annali della Sanità pubblica, 23: 831-844.
27. COLUZZI M. (1963). Le forme di *Anopheles claviger* Meigen indicate con i nomi *missirolii* e *petragnanii* sono due specie riproduttivamente isolate. Accademia Nazionale dei Lincei, Rendiconti della Classe di Scienze fisiche, matematiche e naturali, 32: 1025-1030.
28. COLUZZI M. (1963). Sulla irritabilità al DDT in *Anopheles*. Rivista di Malariologia, 42: 189-222.
29. COLUZZI M. (1964). Osservazioni sperimentali sul comportamento di *Dirofilaria repens* in diversi gruppi di artropodi ematofagi. Parassitologia, 6: 57-62.
30. COLUZZI M., KREMER M. (1964). Contributo alla conoscenza dei culicoidi italiani (Diptera, Ceratopogonidae). Parassitologia, 6: 63-69.
31. COLUZZI M., SACCÀ G., FELICIANGELI D. (1964). Sulla identità delle popolazioni di *Anopheles claviger* nel Medio Oriente. Rivista di Parassitologia, 25: 123-128.
32. WEISER J., COLUZZI M. (1964). *Plistophora culisetae* n. sp. a new Microsporidian (Protozoa Cnidosporidia) in the mosquito *Culiseta longiareolata* (Maquart 1838). Rivista di Malariologia, 43: 51-55.
33. COLUZZI M. (1964). Morphological divergences in the *Anopheles gambiae* complex. Rivista di Malariologia, 43: 197-232.
34. COLUZZI M. (1964). Maintenance of laboratory colonies of *Anopheles* mosquitos. Bulletin of the World Health Organization, 31: 441-443.
35. RIOUX J.A., COLUZZI M., BAIN O., BAUDOUY J.P. (1964). Présence de *Phlebotomus ariasi* Tonnoir, 1921 en Italie du Nord. Bulletin de la Société de Pathologie exotique, 57: 966-971.
36. COLUZZI M. (1964). Experimental infections with *Rubetella fungi* in *Anopheles gambiae* and other mosquitoes. In: Proceedings of the First International Congress of Parasitology, Rome, September 21-26, 1964, I, pp. 592-593.
37. COLUZZI M. (1964). Biological observations on the *Anopheles gambiae* complex. In: Proceedings of the First International Congress of Parasitology, Rome, September 21-26, 1964, II, pp. 902-903.
38. CALLOT J., KREMER M., COLUZZI M. (1965). Nouvelle contribution à l'étude des *Culicoides* (Diptères Ceratopogonides) d'Italie. Parassitologia, 7: 161-171.
39. COLUZZI M., SACCÀ G., FELICIANGELI D. (1965). Il complesso *Anopheles claviger* nella sottoregione Mediterranea. Cahiers O.R.S.T.O.M., Série d'Entomologie Médicale, 3: 97-102.
40. RIOUX J.A., DESCOUS S., COLUZZI M. (1966). *Leptoconops* (L.) *bezzii* Noé, 1905 et *Leptoconops* (L.) *irritans* Noé, 1905 (Diptera, Ceratopogonidae). Cahiers des Naturalistes, Bulletin des Naturalistes Parisiens, 22: 97-100.
41. COLUZZI M. (1966). Osservazioni comparative sul cromosoma X nelle specie A e B del complesso *Anopheles gambiae*. Accademia Nazionale dei Lincei, Rendiconti della Classe di Scienze fisiche, matematiche e naturali, 40: 671-678.
42. COLUZZI M. (1967). Sulla presenza in Italia di *Leptoconops kerteszi* Kieffer (Diptera, Ceratopogonidae). Rivista di Parassitologia, 28: 43-46.
43. COLUZZI M., COLUZZI A. (1967). Su alcune specie di *Aedes* (Diptera, Culicidae) degli Appennini e del Gargano. Rivista di Parassitologia, 28: 47-61.
44. COLUZZI M., SABATINI A. (1967). Cytogenetic observations on species A and B of the *Anopheles gambiae* complex. Parassitologia, 9: 73-88.
45. COLUZZI M., SABATINI A. (1968). Divergenze morfologiche e barriere di sterilità nel complesso *Aedes mariae* (Diptera, Culicidae). Rivista di Parassitologia, 29: 49-70.
46. COLUZZI M., TRABUCCHI R. (1968). Importanza dell'armatura bucco-faringea in *Anopheles* e *Culex* in relazione alle infezioni con *Dirofilaria*. Parassitologia, 10: 47-59.
47. COLUZZI M., SABATINI A. (1968). Cytogenetic observations on species C of the *Anopheles gambiae* complex. Parassitologia, 10: 155-165.
48. COLUZZI M., SABATINI A. (1968). Cytogenetic observations on species C, *merus* and *melas* of the *Anopheles gambiae* complex. Cahiers ORSTOM, Série d'Entomologie Médicale, 6: 211-213.
49. COLUZZI M. (1968). Cromosomi politenici delle cellule nutrici ovariche nel complesso *gambiae* del genere *Anopheles*. Parassitologia, 10: 179-183.
50. COLUZZI M. (1968). Nuove segnalazioni di culicidi in Sicilia (Diptera, Nematocera). Bollettino della Società Entomologica Italiana, 98: 126-128.
51. COLUZZI M., COLUZZI A. (1969). Incroci tra popolazioni di *Anopheles labranchiae* e *Anopheles atroparvus*. Parassitologia, 11: 108-109.
52. COLUZZI M., SABATINI A. (1969). Nuove segnalazioni per l'Italia di specie del genere *Culicoides* (Diptera, Ceratopogonidae). Parassitologia, 11: 109-110.
53. COLUZZI M., SABATINI A. (1969). Cytogenetic observations on the salt water species, *Anopheles merus* and *Anopheles melas* of the *gambiae* complex. Parassitologia, 11: 177-187.
54. COLUZZI M. (1970). Sibling species in *Anopheles* and their importance in malariology. Miscellaneous Publications of the Entomological Society of America, 7: 63-77.
55. BULLINI L., COLUZZI M., GIRONI A.M., MORELLINI M. (1970). Phosphoglucomutase polymorphism in *Aedes aegypti*. Parassitologia, 12: 27-30.
56. COLUZZI M., CANCRINI G., DI DECO M. (1970). The polytene chromosomes of *Anopheles superpictus* and relationships with *Anopheles stephensi*. Parassitologia, 12: 101‑112.
57. BULLINI L., GIRONI A.M., BIANCHI BULLINI A.P., COLUZZI M. (1970). Further observations on phosphoglucomutase polymorphism in *Aedes aegypti*. Parassitologia, 12: 113-117.
58. COLUZZI M., GIRONI A.M., MUIR D.A. (1970). Ulteriori esperimenti d'incrocio tra le forme del complesso *mariae* del genere *Aedes*. Parassitologia, 12: 119-123.
59. COLUZZI M. (1971). Problèmes théoriques et pratiques liés à l'élevage et à la production de masse des Culicides. Annales de Parasitologie humaine et comparée, 46: 91-101.
60. COLUZZI M., BULLINI L. (1971). Enzyme variants as markers in the study of pre-copulatory isolating mechanisms. Nature, 231: 455-456.
61. COLUZZI M., BULLINI L., BIANCHI BULLINI A.P. (1971). Phosphoglucomutase polymorphism in *Aedes phoeniciae* Coluzzi & Sabatini of the *Aedes mariae* complex (Diptera, Culicidae). Bulletin of Entomological Research, 61: 327-330.
62. BULLINI L., COLUZZI M., CANCRINI G., SANTOLAMAZZA C. (1971). Multiple phosphoglucomutase alleles in *Anopheles stephensi*. Heredity, 26: 475-478.
63. COLUZZI M., CANCRINI G. (1971). I cromosomi salivari di *Anopheles plumbeus* Stephens. Parassitologia, 13: 145-149.
64. COLUZZI M., GIRONI A.M. (1971). Osservazioni preliminari sulla selezione di ceppi di *Aedes aegypti* sensibili e resistenti all'infezione da *Dirofilaria repens*. Parassitologia, 13: 151-152.
65. COLUZZI M., BULLINI L., BIANCHI BULLINI A.P. (1971). Phosphoglucomutase (PGM) allozymes in two forms of the *mariae* complex of the genus *Aedes*. Biochemical Genetics, 5: 253-255.
66. BRYAN J.H., COLUZZI M. (1971). Cytogenetic observations on *Anopheles farauti* Laveran. Bulletin of the World Health Organization, 45: 266-267.
67. KREMER M., COLUZZI M. (1971). Description de *Culicoides malevillei* n.sp. (Diptera, Ceratopogonidae). Parassitologia, 13: 415-419.
68. BULLINI L., COLUZZI M., BIANCHI BULLINI A.P., BLEINER G. (1971). Phosphoglucomutase polymorphism in *Culex pipiens* (Diptera, Culicidae). Parassitologia, 13: 439-443.
69. COLUZZI M., CANCRINI G., DI DECO M. (1971). Esperimenti d'incrocio tra *Anopheles stephensi* e *Anopheles superpictus*. Parassitologia, 13: 445-448.
70. COLUZZI M. (1972). Inversion polymorphism and adult emergence in *Anopheles stephensi*. Science, 176: 59-60.
71. BULLINI L., GIRONI A.M., BIANCHI BULLINI A.P., COLUZZI M. (1972). Phosphoglucomutase gene in *Aedes aegypti*: a fourth allele and preliminary linkage data. Biochemical genetics, 7: 41-44.
72. BULLINI L., COLUZZI M., BIANCHI BULLINI A.P. (1972). Studi genetici sulla fosfoglucomutasi nei Ditteri Culicidi. Atti dell’Associazione Genetica Italiana, 17: 43-45.
73. BULLINI L., COLUZZI M. (1972). I sistemi gene-enzima nella lotta genetica. Parassitologia, 14: 67-70.
74. BULLINI L., COLUZZI M. (1972). Natural selection and genetic drift in protein polymorphism. Nature, 239: 160-161.
75. COLUZZI M., CANCRINI G., DI DECO M. (1972). Polimorfismo cromosomico e lunghezza del'uovo in *Anopheles stephensi*. Parassitologia, 14: 261-266.
76. BULLINI L., COLUZZI M., BIANCHI BULLINI A.P., RENNA L. (1972). Stability of frequencies of phosphoglucomutase alleles in *Culex pipiens* breeding in ecologically different environments. Accademia Nazionale dei Lincei, Rendiconti della Classe di Scienze fisiche, matematiche e naturali, 53: 154-157.
77. COLUZZI M., PETRARCA V. (1973). Aspirator with paper cup for collecting mosquitoes and other insects. Mosquito News, 33: 249-250.
78. CLASTRIER J., COLUZZI M. 1973. *Leptoconops* (*Leptoconops*) *bezzii* (Noé, 1905) et *Leptoconops* (*Leptoconops*) *noei* n.sp. (Diptera, Ceratopogonidae). Parassitologia, 15: 47-77.
79. COLUZZI M., DI DECO M., CANCRINI G. (1973). Chromosomal inversions in Anopheles stephensi. Parassitologia, 15: 129-136.
80. BULLINI L., COLUZZI M., BIANCHI BULLINI A.P., CANCRINI G. 1973. A new phosphoglucomutase (PGM) allele in *Aedes aegypti* (Diptera, Culicidae). Parassitologia, 15: 141-144.
81. COLUZZI M. 1973. Laboratory and field observations on inversion polymorphism in Anopheline mosquitoes. In: Proceedings of the 9th International Congress on Tropical Medicine and Malaria, Athens, October 14-21, 1973, pp.250-251.
82. COLUZZI M., DI DECO M., CANCRINI G. (1973). Ulteriori osservazioni sulla lunghezza dell'uovo in *Anopheles stephensi* in relazione al polimorfismo cromosomico. Parassitologia, 15: 213-215.
83. BULLINI L., COLUZZI M. 1973. Electrophoretic studies of gene-enzyme systems in mosquitoes (Diptera, Culicidae). Parassitologia, 15: 221-248.
84. COLUZZI M., DI DECO M., GIRONI A.M. (1974). Embryonic diapause in *Aedes mariae* and changes in the ovoposition behaviour induced by photoperiod. In: Proceedings of the 3rd International Congress of Parasitology, Munich, August 1974, 2: 902-903.
85. COLUZZI M., SABATINI A., PETRARCA V. (1974). Polimorfismi cromosomici e loro significato adattativo nel complesso *gambiae* (genere *Anopheles*). Parassitologia, 16: 107‑109.
86. COLUZZI M., DI DECO M.A. (1974). Cage experiments on homospecific and heterospecific matings with females of *Anopheles stephensi* carriers of different inversion karyotypes. Accademia Nazionale dei Lincei, Rendiconti della Classe di Scienze fisiche, matematiche e naturali, 57: 683-689.
87. COLUZZI M., SABATINI A., BULLINI L., RAMSDALE C. (1974). Nuovi dati sulla distribuzione delle specie del complesso mariae del genere *Aedes*. Rivista di Parassitologia, 35: 321-330.
88. COLUZZI M., CANCRINI G. (1974). Genetica della suscettibilità di *Aedes aegypti* a *Dirofilaria repens*. Parassitologia, 16: 239-256.
89. BULLINI M., COLUZZI M., BIANCHI BULLINI A.P. (1974). Un nuovo approccio allo studio della ricettività sessuale nelle femmine di *Culex pipiens*. Atti dell’Associazione Genetica Italiana, 19: 24-29.
90. BULLINI L., CANCRINI G., BIANCHI BULLINI A.P., COLUZZI M. (1974). Polimorfismo bilanciato in una popolazione di laboratorio di *Anopheles stephensi* (Diptera, Culicidae). Atti dell’Associazione Genetica Italiana, 20: 69-72.
91. BULLINI L., COLUZZI M., BIANCHI BULLINI A.P. (1974). Competitività sessuale in *Culex pipiens* in esperimenti di lancio con ceppi marcati per mezzo di varianti enzimatici. In:Atti del X Congresso Nazionale Italiano di Entomologia, Sassari, 20-25 maggio 1974, pp.273-274.
92. RAMSDALE C.D., COLUZZI M. (1975). Studies on the infectivity of tropical African strains of *Plasmodium falciparum* to some southern European vectors of malaria. Parassitologia, 17: 39-48.
93. CANCRINI G., COLUZZI M., BALBO T., GALLO M.G. (1975). Variazioni stagionali della microfilariemia ed effetto della temperatura ambientale in cani parassitati da *Dirofilaria repens*. Parassitologia, 17: 75-82.
94. COLUZZI M., DI DECO M.A., GIRONI A.M. (1975). Influenza del fotoperiodo sulla scelta del luogo di ovideposizione in *Aedes mariae* (Diptera, Culicidae). Parassitologia, 17: 121-130.
95. COLUZZI M., DI DECO M.A., PETRARCA V. (1975). Propensione al pasto di sangue in condizioni di laboratorio e polimorfismo cromosomico in *Anopheles stephensi*. Parassitologia, 17: 137-143.
96. COLUZZI M., SABATINI A., PETRARCA V. (1975). Chromosomal investigations on species A and B of the *Anopheles gambiae* complex in the Garki district (Kano State, Nigeria). Results of species identifications from 1971 to 1974. World Health Organization MPD/TN/75.1 pp. 16-25.
97. WHITE G.B., COLUZZI M., ZAHAR A.R. (1975). Review of cytogenetic studies on Anopheline vectors of malaria. World Health Organization MAL/ 75.849 - VBC/75.538. pp. 1-35.
98. DE ZULUETA J., RAMSDALE C., COLUZZI M. (1975). Receptivity to malaria in Europe. Bulletin of the World Health Organization, 52: 109-111.
99. BULLINI L., COLUZZI M., BIANCHI BULLINI A.P. (1976). Biochemical variants in the study of multiple insemination in *Culex pipiens* L. (Diptera, Culicidae). Bulletin of Entomological Research, 65: 683-685.
100. CANCRINI G., DI DECO M.A., COLUZZI M., BIANCHI BULLINI A.P., CIANCHI R., BULLINI L. (1976). Ricerche sull'associazione tra riordinamenti cromosomici e sistemi gene-enzima in *Anopheles stephensi*. Bollettino di Zoologia, 43: 352-353.
101. COLUZZI M., BIANCHI BULLINI A.P., BULLINI L. (1976). Speciazione nel complesso *mariae* del genere *Aedes* (Diptera,Culicidae). Atti dell’Associazione Genetica Italiana, 21: 218-223.
102. PETRARCA V., CIANCHI R., SABATINELLI G., BIANCHI BULLINI A.P., COLUZZI M., BULLINI L. (1976). Alcuni dati sul differenziamento morfologico, ecologico, etologico e genetico di popolazioni urbane e rurali di *Culex pipiens* nel Lazio. Bollettino di Zoologia, 43: 394-395.
103. GATTI M., PIMPINELLI S., SANTINI G., COLUZZI M., BULLINI L. (1976). Lo studio dell'eterocromatina nella sistematica entomologica. In:Atti del l’XI Congresso Nazionale Italiano di Entomologia, Portici-Sorrento, 10-15 maggio 1976, pp 123-134.
104. BULLINI L., COLUZZI M., BIANCHI BULLINI A.P. (1976). Lotta genetica nelle zanzare mediante lancio di maschi non conspecifici e di ibridi sterili. Atti dell’XI Congresso Nazionale Italiano di Entomologia, Portici-Sorrento, 10-15 maggio 1976, pp. 339-342.
105. GATTI M., SANTINI G., PIMPINELLI S., COLUZZI M. (1977). Fluorescence banding techniques in the identification of sibling species of the *Anopheles gambiae* complex. Heredity, 38: 105-108.
106. COLUZZI M. (1977). Lotta chimica e biologia dei vettori. Parassitologia, 19: 169-173.
107. COLUZZI M., SABATINI A., PETRARCA V., BIANCHI BULLINI A.P., BULLINI L. (1977). Differenziamento genetico nelle specie del complesso *Anopheles gambiae*. Atti dell'Associazione Genetica Italiana, 22: 147-149.
108. COLUZZI M., SABATINI A., PETRARCA V., DI DECO M.A. 1977. Behavioural divergences between mosquitoes with different inversion karyotypes in polymorphic populations of the *Anopheles gambiae* complex. Nature, 266: 832-833.
109. BULLINI L., COLUZZI M., (1978). Applied and theoretical significance of electrophoretic studies in mosquitoes (Diptera, Culicidae). Parassitologia, 20: 7-21.
110. CIANCHI R., URBANELLI S., COLUZZI M., BULLINI L. (1978). Genetic distance between two sibling species of the *Aedes mariae* complex (Diptera, Culicidae). Parassitologia, 20: 39-46.
111. COLUZZI M. (1978). Aspetti attuali della lotta contro artropodi di importanza sanitaria in Italia. Rivista di Parassitologia, 39: 161-166.
112. COLUZZI M., SABATINI A., PETRARCA V., DI DECO M.A. (1978). Inversion polymorphism and house resting behaviour in populations of the *Anopheles gambiae* complex. In: Proceedings of the Fourth International Congress of Parasitology, Short Communications, Section G, Warszawa, August 1978, p. 6.
113. COLUZZI M., DI DECO M.A., PETRARCA V., SABATINI A. (1978). Sibling species of the *Anopheles gambiae* complex and urban malaria in West African forest areas. In: Proceedings of the Fourth International Congress of Parasitology, Short Communications, Section G, Warszawa, August 1978, pp.6-7.
114. DI DECO M.A., CANCRINI G., COLUZZI M., BIANCHI BULLINI A.P., CIANCHI R., BULLINI L. (1978). Linkage studies between chromosome inversions and enzyme loci in the mosquito *Anopheles stephensi*. Heredity, 40: 457-458.
115. CANCRINI G., MANTOVANI A., COLUZZI M. (1979). Infestazione sperimentale del gatto con *Dirofilaria repens* di origine canina. Parassitologia, 21: 89-90.
116. COLUZZI M., SABATINI A., PETRARCA V., DI DECO M.A. (1979). Chromosomal differentiation and adaptation to human environments in the *Anopheles gambiae* complex. Transactions of the Royal Society of Tropical Medicine and Hygiene, 73: 483-497.
117. PETRARCA V., POZZESSERE S., COLUZZI M. (1979). Infezioni da *Coelomomyces* spp. in *Anopheles gambiae* s.l. in Nigeria. Parassitologia, 21: 121-122.
118. SABATINI A., COLUZZI M., DI DECO M.A. (1979). Variabilità cromosomica in popolazioni di *Anopheles superpictus* dell'Italia meridionale. Parassitologia, 21: 124-125.
119. BIANCHI BULLINI A.P., CIANCHI R., SABATINI A., COLUZZI M., BULLINI L. (1980). Ricerche elettroforetiche su specie paleartiche del complesso *Anopheles maculipennis* (Diptera, Culicidae). In: Atti del XII Congresso Nazionale Italiano di Entomologia, Roma, 1980, II, pp. 255-259.
120. BONACCORSI S., SANTINI G., GATTI F., PIMPINELLI S., COLUZZI M. (1980). Intraspecific polymorphism of sex chromosome heterochromatin in two species of the *Anopheles gambiae* complex. Chromosoma, 76: 57-64.
121. BULLINI L., BIANCHI BULLINI A.P., CIANCHI R., SABATINI A., COLUZZI M. (1980). Tassonomia biochimica del complesso *Anopheles maculipennis*. Parassitologia, 22: 290-292.
122. CIANCHI R., SABATINI A., COLUZZI M., BULLINI L. (1980). Divergenza genetica tra due specie gemelle del genere *Anopheles*: *Anopheles claviger* e *Anopheles petragnanii* (Diptera, Culicidae). In: Atti del XII Congresso Nazionale Italiano di Entomologia, Roma, 1980, II, pp.261-263.
123. CIANCHI R., URBANELLI S., SABATINI A., COLUZZI M., TORDI M.P., BULLINI L. (1980). Ricerche elettroforetiche su specie dei sottogeneri *Ochlerotatus* e *Finlaya* (genere *Aedes*): aspetti tassonomici ed evolutivi (Diptera, Culicidae). In:Atti del XII Congresso Nazionale Italiano di Entomologia, Roma, 1980, II, pp.265-267.
124. CIANCHI R., URBANELLI S., SABATINI A., COLUZZI M., TORDI M.P., BULLINI L. (1980). Due entità riproduttivamente isolate sotto il nome di *Aedes caspius* (Diptera, Culicidae). In: Atti del XII Congresso Nazionale Italiano di Entomologia, Roma, 1980, II, pp. 269-272.
125. URBANELLI S., COLUZZI M., PETRARCA V., BULLINI L. (1980). Differenziamento genetico in popolazioni italiane di *Culex pipiens* (Diptera, Culicidae). In: Atti del XII Congresso Nazionale Italiano di Entomologia, Roma, 1980, II, pp. 273-280.
126. COLUZZI M., CONCETTI A., ASCOLI F. (1980). Armature cibarica e faringea nelle zanzare (Diptera, Culicidae) ed emolisi del pasto di sangue. In: Atti del XII Congresso Nazionale Italiano di Entomologia, Roma, 1980, II, pp.391-393.
127. COLUZZI M. (1980). Recent advances in the cytogenetic study of Afrotropical malaria vectors. Rendiconti Accademia Nazionale delle Scienze detta dei XL, Memorie di Scienze Fisiche e Naturali, IV: 205-210.
128. COLUZZI M., COLUZZI A. (1980). Rischio di una ripresa della trasmissione della malaria in Calabria e problemi connessi al controllo dei vettori. Rivista di Parassitologia, 41: 117-125.
129. DI DECO M.A., PETRARCA V., VILLANI F., COLUZZI M. (1980). Polimorfismo cromosomico da inversioni paracentriche ed eccesso degli eterocariotipi in ceppi di *Anopheles* allevati in laboratorio. Parassitologia, 22: 304-306.
130. DI DECO M.A., PETRARCA V., VILLANI F., COLUZZI M. (1980). Recombination and linkage disequilibria between chromosome-2 inversions in *Anopheles gambiae* s.s. In: Proceedings of the Third European Multicolloquium of Parasitology, Cambridge, England, September 7-13, 1980, p. 87.
131. PETRARCA V., DORTLEMEZ G., SABATINI A., COLUZZI M. (1980). Osservazioni sui cromosomi politenici di *Anopheles sacharovi* della Turchia e dell'Italia. Parassitologia, 22: 336-338.
132. PETRARCA V., ORECCHIA P., COLUZZI M. (1980). Osservazioni sulla biologia di *Plagiorchis elegans* (Rudolphi,1802). Parassitologia, 22: 338-339.
133. PETRARCA V., SABATINELLI G., COLUZZI M. (1980). Significato di alcune differenze biometriche in diverse popolazioni del complesso *Culex pipiens*. Parassitologia, 22: 340-342.
134. BRYAN J.H., DI DECO M.A., PETRARCA V., COLUZZI M. (1981). Polimorfismo cromosomico di *Anopheles gambiae* s.s. nella Gambia e zone limitrofe. Parassitologia, 23: 132-136.
135. BRYAN J.H., PETRARCA V., DI DECO M.A., COLUZZI M. (1981). Osservazioni su *Anopheles melas* nella Gambia e zone limitrofe. Parassitologia, 23: 137-139.
136. BULLINI L., URBANELLI S., COLUZZI M. (1981). Polimorfismo per la glutammico-ossalacetico transaminasi in *Culex pipiens*. Parassitologia, 23: 142-145.
137. CIANCHI R., SABATINI A., BULLINI L., COLUZZI M. (1981). Differenziazione morfologica e genetica nei complessi *Anopheles maculipennis* e *Anopheles claviger*. Parassitologia, 23: 158-163.
138. COLUZZI M., DALLAI R., INSOM E. (1981). Morfologia e funzione delle armature cibariche nelle zanzare. Parassitologia, 23: 164-168.
139. COLUZZI M., DUNBAR R.W. (1981). Cytogenetics of vector-hosts. In: Review of Advances in Parasitology, Warzsawa, 1981, pp. 775-783.
140. PACI P., LEONCINI F., MAZZOTTA F., MILO D., AMADUCCI L., FRATIGLIONI L., CAMPANELLA G., CIUFOLINI M.G., NICOLETTI L., VERANI P., COLUZZI M., SABATINELLI G., BALDUCCI M. (1981). Infezioni acute del SNC nella provincia di Firenze: studio epidemiologico ed ecologico. In: La regione Toscana per la lotta contro le malattie infettive. Atti del II Convegno Regionale, Firenze, 8-9 Maggio 1981, pp. 205-216.
141. SABATINI A., COLUZZI M., LANFRANCHI P. (1981). Sulla presenza in Italia settentrionale di *Aedes* (*Ochlerotatus*) *cantans*. Parassitologia, 23: 244-245.
142. SABATINI A., TERRANOVA F., CIANCHI R., COLUZZI M. (1981). Ricerche sull'anofelismo delle fiumare della costa ionica calabrese. Parassitologia, 23: 245-249.
143. URBANELLI S., CIANCHI R., PETRARCA V., SABATINELLI G., COLUZZI M., BULLINI L. (1981). Adattamento all'ambiente urbano della zanzara *Culex pipiens* (Diptera, Culicidae). In: Atti S.IT.E. (Ecologia), I, pp. 305-316.
144. BRYAN J.H., DI DECO M.A., PETRARCA V., COLUZZI M. (1982). Inversion polymorphism and incipient speciation in *Anopheles gambiae* s.str. in The Gambia, West Africa. Genetica, 59: 167-176.
145. CAMBOURNAC F.J.C., PETRARCA V., COLUZZI M. (1982). *Anopheles arabiensis* in the Cape Verde archipelago. Parassitologia, 24: 265-267.
146. COLUZZI M., CONCETTI A., ASCOLI F. (1982). Effect of cibarial armature of mosquitoes (Diptera, Culicidae) on blood-meal haemolysis. Journal of Insect Physiology, 28: 885-888.
147. VERANI P., CIUFOLINI M.G., NICOLETTI L., BALDUCCI M., SABATINELLI G., COLUZZI M., PACI P., AMADUCCI L. (1982). Studi ecologici ed epidemiologici del virus Toscana, un arbovirus isolato da flebotomi. Annali dell'Istituto Superiore di Sanità, 18: 397-400.
148. CIANCHI R., PETRARCA V., COLUZZI M., BULLINI L. (1983). Identificazione cromosomica e biochimica dei membri del complesso *Anopheles gambiae* (Diptera, Culicidae). In: Atti del XIII Congresso Nazionale Italiano d Entomologia, Sestrière, Torino, 1983, pp. 499-503.
149. SABATINI A., COLUZZI M., BOCCOLINI D. (1983). Distribuzioni non casuali nell'ambiente domestico di varianti cromosomici di *Anopheles superpictus*. Parassitologia, 25: 348-349.
150. TOURÉ Y.T., PETRARCA V., COLUZZI M. (1983). Répartition géographique et polymorphisme chromosomique des membres du complexe *Anopheles gambiae* au Mali. In: Proceedings of the 2nd International Conference on Malaria and Babebiosis, Annecy, France, September 19-22, 1983, p. 72.
151. TOURÉ Y.T., PETRARCA V., COLUZZI M. (1983). Nuove entità del complesso *Anopheles gambiae* in Mali. Parassitologia, 25: 367-370.
152. DE ZULUETA J., RAMSDALE C., CIANCHI R., BULLINI L., COLUZZI M. (1983). Observations on the taxonomic status of *Anopheles sicaulti*. Parassitologia, 25: 73-92.
153. TOURÉ Y.T., PETRARCA V., COLUZZI M. (1984). The *Anopheles gambiae* complex in Mali. In: Proceedings of the XI International Congress for Tropical Medicine and Malaria, Calgary, Canada, September 16-22, 1984, p. 157.
154. COLUZZI M. (1984). Heterogeneities of the malaria vectorial system in tropical Africa and their significance in malaria epidemiology and control. Bulletin of the World Health Organization, 62: 107-113.
155. SABATINI A., COLUZZI M., BOCCOLINI D. (1984). Excess of inversion heterozygotes in indoor resting samples of *Anopheles superpictus*. In: Proceedings of the XI International Congress for Tropical Medicine and Malaria, Calgary, Canada, September 16‑22, 1984, p. 130.
156. PACI P., BALDUCCI M., VERANI P., COLUZZI M., AMADUCCI L., LEONCINI F., NICOLETTI L., CIUFOLINI M.G., FRATIGLIONI L. (1984). Toscana virus, a new *Phlebotomus*-transmitted virus isolated in Italy. In: Proceedings of the International Congress for Infectious Diseases, pp. 35-39.
157. COLUZZI M., PETRARCA V., DI DECO M.A. (1985). Chromosomal inversion intergradation and incipient speciation in *Anopheles gambiae*. Bollettino di Zoologia, 52: 45‑63.
158. COLUZZI M., SABATINI A., MAJORI G. (1985). Le zanzare nelle zone urbane. In: Atti dell'Accademia Nazionale Italiana di Entomologia, pp. 123-131.
159. RISHIKESH N., DI DECO M.A., PETRARCA V., COLUZZI M. (1985). Seasonal variations in indoor resting *Anopheles gambiae* and *Anopheles arabiensis* in Kaduna, Nigeria. Acta Tropica, 42: 165-170.
160. MUNSTERMAN L.E., MARCHI A., SABATINI A., COLUZZI M. (1985). Polytene chromosomes of *Orthopodomyia pulcripalpis* (Diptera, Culicidae). Parassitologia, 27: 267‑277.
161. BOCCOLINI D., SABATINI A., COLUZZI M. (1986). Valore diagnostico del numero dei rami delle setole antepalmate per l'identificazione delle specie italiane del complesso *Anopheles maculipennis*. Annali dell'Istituto Superiore di Sanità, 22: 201-204.
162. COLUZZI M., SABATINI A. (1986). Ruolo della competizione nella distribuzione di vettori di malaria in Italia. Annali dell'Istituto Superiore di Sanità, 22: 205-208.
163. TOURÉ Y.T., PETRARCA V., COLUZZI M. (1986). Esame comparativo dei tassi di infezione con sporozoiti e filarie in diverse forme del complesso *Anopheles gambiae* in un villaggio del Mali. Annali dell'Istituto Superiore di Sanità, 22: 215-218.
164. BRYAN J.H., PETRARCA V., DI DECO M.A., COLUZZI M. (1986). Comportamento di puntura e di riposo di *Anopheles melas* e dei suoi varianti cromosomici. Annali dell'Istituto Superiore di Sanità, 22: 229-232.
165. COLUZZI M., PETRARCA V., DI DECO M.A., TOURÉ Y.T. (1986). Malaria vectors and transmission. In: Proceedings of the Conference on Malaria in Africa - Practical Considerations on Malaria Vaccines and Clinical Trials, Washington D.C., U.S.A., December 1-4, 1986, pp. 69-72.
166. ESPOSITO F., LOMBARDI S., TOURÉ Y.T., ZAVALA F., COLUZZI M. (1986). Field observations on the use of anti-sporozoite monoclonal antibodies for determination of infection rates in malaria vectors. Parassitologia, 28: 69-77.
167. TOURÉ Y.T., COLUZZI M. (1986). Barrière de tulle moustiquaire pour l'échantillonnage des Culicidés zoophiles. Parassitologia, 28: 79-82.
168. ESPOSITO F., LOMBARDI S., MODIANO D., ZAVALA F., REEME J., LAMIZANA L., COLUZZI M., NUSSENZWEIG R.S. (1986). Immunity to *Plasmodium* sporozoites: recent advances and applications to field research. Parassitologia, 28: 101-105.
169. LOMBARDI S., ESPOSITO F., ZAVALA F., LAMIZANA L., ROSSI P., SABATINELLI G., NUSSENZWEIG R.S., COLUZZI M. (1986). CS antigen localization in malaria vectors: hypothetical refractoriness to transmission observed in the field. Parassitologia, 28: 113-116.
170. PETRARCA V., NUGUD A.D., ELKARIM AHMED M.A., HARIDI A.M., ABDELNUR O.M., COLUZZI M. (1986). Dati preliminari sul complesso *Anopheles gambiae* in Sudan. Parassitologia, 28: 304-306.
171. ROBERT V., PETRARCA V., CARNEVALE P., COLUZZI M. (1986). Le particularisme de la transmission du paludisme dans la zone rizicole de la Vallée du Kou (Burkina Faso); l'apport de l'étude cytogénétique des vecteurs à l'épidémiologie. Parassitologia, 28: 327-329.
172. SABATINI A., BOCCOLINI D., TOURÉ Y.T., PETRARCA V., COLUZZI M. (1986). Polimorfismi cromosomici e morfologici in *Anopheles rufipes*. Parassitologia, 28: 337-339.
173. CIANCHI R., SABATINI A., BOCCOLINI D., BULLINI L., COLUZZI M. (1987). Electrophoretic evidence of reproductive isolation between sympatric populations of *Anopheles melanoon* and *Anopheles subalpinus*. In: Proceedings of the 3rd International Conference on Malaria and Babesiosis, Annecy, France, September 7-11, 1987, p. 156.
174. LOMBARDI S., ESPOSITO F., LENSEN A.H.W., PONNUDURAI T., COLUZZI M. (1987). Antibodies contained in the mosquito bloodmeal can be detected in the hemolymph. In: Proceedings of the 3rd International Conference on Malaria and Babesiosis, Annecy, France, September 7-11, 1987, p. 159.
175. TOURÉ Y.T., PETRARCA V., COLUZZI M., (1987). Patterns of geographical and seasonal distributions of the "Mopti" karyotypes of *Anopheles gambiae* in Mali. In: Proceedings of the 3rd International Conference on Malaria and Babesiosis, Annecy, France, September 7-11, 1987, p. 172.
176. LOMBARDI S., ESPOSITO F., ZAVALA F., LAMIZANA L., ROSSI P., SABATINELLI G., NUSSENZWEIG R.S., COLUZZI M. (1987). Detection and anatomical localization of *P. falciparum* circumsporozoite protein and sporozoites in the Afrotropical malaria vector *Anopheles gambiae* s.l. American Journal of Tropical Medicine and Hygiene, 37: 491-494.
177. MAJORI G., SABATINELLI G., COLUZZI M. (1987). Efficacy of permethrin-impregnated curtains for malaria vector control. Medical and Veterinary Entomology, 1: 185-192.
178. PETRARCA V., VERCRUYSSE J., COLUZZI M. (1987). Observations on the *Anopheles gambiae* complex in the Senegal River Basin, West Africa. Medical and Veterinary Entomology, 1: 303-312.
179. RALISOA RANDRIANASOLO B.O., COLUZZI M. (1987). Genetical investigations on zoophilic and exophilic *Anopheles arabiensis* from Antanarivo area (Madagascar). Parassitologia, 29: 93-97.
180. COLUZZI M (1988). Maitriser l’évolution du paludisme sur les Hautes-Terres de Madagascar. Interview by G. Ramahandridona. Ny Malagasy 1: 18-21.
181. BRYAN J.H., PETRARCA V., DI DECO M.A., COLUZZI M. (1987). Adult behaviour of members of the *An. gambiae* complex in The Gambia with special reference to *An. melas* and its chromosomal variants. Parassitologia, 29: 221-249.
182. ROBERT V., CARNEVALE P., OUEDRAOGO V., PETRARCA V., COLUZZI M. (1988). La transmission du paludisme humaine et les Culicidae dans un village de savane du sud-ouest du Burkina Faso. Annales de la Société Belge de Médecine Tropicale, 68: 107‑121.
183. ESPOSITO F., LOMBARDI S., MODIANO D., ZAVALA F., REEME J., LAMIZANA L., COLUZZI M., NUSSENZWEIG R.S. (1988). Prevalence and levels of antibodies to the circumsporozoite protein of *Plasmodium falciparum* in an endemic area and their relationship to resistence against malaria infection. Transactions of the Royal Society of Tropical Medicine, 82: 827-832.
184. VERANI P., CIUFOLINI M.G., CACIOLLI S., RENZI A., NICOLETTI L., SABATINELLI G., BARTOLOZZI D., VOLPI G., AMADUCCI L., COLUZZI M., PACI P., BALDUCCI M. (1988). Ecology of viruses isolated from sand flies in Italy and characterization of a new Phlebovirus (Arbia virus). American Journal of Tropical Medicine and Hygiene, 38: 433-439.
185. AKOGBETO M., DI DECO M.A., ROMANO R., COLUZZI M. (1988). Analisi comparativa del ruolo vettore di *Anopheles gambiae* e *An. melas* nella zona lagunare costiera del Benin. Parassitologia, 30(S): 7-8.
186. CANCRINI G., SUN YANCHANG., DELLA TORRE A., COLUZZI M. (1988). Influenza della temperatura sullo sviluppo larvale di *Dirofilaria repens* in diverse specie di zanzare. Parassitologia, 30(S): 38.
187. ROBERT V., PETRARCA V., CARNEVALE P., OVAZZA L., COLUZZI M. (1989). Analyse citogénétique du complexe *Anopheles gambiae* dans la région de Bobo-Dioulasso (Burkina Faso). Annales de Parasitologie Humaine et Comparée, 64: 290-311.
188. COLUZZI M., PETRARCA V., DI DECO M.A. (1989). Adaptive chromosomal variation in the *Anopheles gambiae* complex. In: Proceedings of the 2nd Congress of the European Society for Evolutionary Biology, Rome, September 25-29, 1989, Abs. s1, p. 19.
189. DELLA TORRE A., CUERVO AYALDE A.C., PETRANGELI G., COLUZZI M. (1989). Experimental introgression of paracentric inversions between *Anopheles arabiensis* and *Anopheles gambiae* s.s. In: Proceedings of the 2nd Congress of the European Society for Evolutionary Biology Rome, September 25-29, 1989, Abs. w2, p. 21.
190. SABATINI A., COLUZZI M., BOCCOLINI D. (1989). Field studies on inversion polymorphism in *Anopheles superpictus* from Southern Italy. Parassitologia, 31: 69-87.
191. COOSEMANS M., PETRARCA V., BARUTWANAYO M., COLUZZI M. (1989). Species of the *Anopheles gambiae* complex and chromosomal polymorphism in a rice-growing area of the Rusizy valley (Republic of Burundi). Parassitologia, 31: 113-122.
192. CANCRINI G., TASSI P., COLUZZI M. (1989). Ivermectin against larval stages of *Dirofilaria repens* in dogs. Parassitologia, 31: 177-182.
193. CANCRINI G., MATTIUCCI S., D'AMELIO S., GENCHI C., COLUZZI M. (1989). Genetic characterization of *Dirofilaria repens* and *D. immitis* by electrophoretic analysis of gene-enzyme systems. Parassitologia, 31: 189-196.
194. ROBERT V., PETRARCA V., CARNEVALE P., ZOULANI A., COLUZZI M. (1990). Analyse cytogénétique du complexe *Anopheles gambiae* dans un village du Sud-Est du Burkina Faso. Genetics Selection Evolution, 22: 161-174.
195. AKOGBETO M., DI DECO M.A., MUKABAYIRE O., COLUZZI M. (1990). The *Anopheles gambiae* complex in Togo and Benin: species identification and inversion polymorphisms. In: Proceedings of the ICOPA VII, Paris, August 20-24, 1990. Bulletin de la Société Francaise de Parasitologie, Abs. 10A1, p. 1128.
196. AKOGBETO M., ROMANO R., COLUZZI M. (1990). Increase of *Plasmodium falciparum* transmission potential in the coastal areas of Benin. In: Proceedings of the ICOPA VII, Paris, August 20-24, 1990. Bulletin de la Société Francaise de Parasitologie, Abs 10C2 , p. 1168.
197. FONTENILLE D., CAMPBELL C., COLUZZI M., LEPERS J.P., COULANGES P. (1990). Comparaison de deux facies de transmission du paludisme à Madagascar. In: Proceedings of the ICOPA VII, Paris, August 20-24, 1990. Bulletin de la Société Francaise de Parasitologie, Abs. 10C18, p. 1176.
198. SUZZONI-BLATGER J., CIANCHI R., BULLINI L., COLUZZI M. (1990). Le complexe *maculipennis*: critères morphologiques et enzymatiques de détermination. Annales de Parasitologie Humaine et Comparée, 65: 37-40.
199. FONTENILLE D., LEPERS J-P., CAMPBELL G.H., COLUZZI M., RAKOTO-ARIVONY I., COULANGES P. (1990). Malaria transmission and vector biology in Manarintsoa, high plateaux of Madagascar. American Journal of Tropical Medicine and Hygiene, 43: 107-115.
200. CARRARA G.C., PETRARCA V., NIANG M., COLUZZI M. (1990). *Anopheles pharoensis* and transmission of *Plasmodium falciparum* in the Senegal River delta, West Africa. Medical and Veterinary Entomology, 4: 421-424.
201. FONTENILLE D., LEPERS J.P., CAMPBELL G.H., RAKOTOARIVONY I., COLUZZI M., COULANGES P. (1990). Les vecteurs du paludisme et leur rôle dans la transmission, à Manarintsoa sur les hauts-plateaux de Madagascar, de 1988 à 1990. Archives de l'Institut Pasteur de Madagascar, 57: 335-368.
202. COLUZZI M., PETRARCA V., DI DECO M.A. (1990). Variazioni cromosomiche in *Anopheles gambiae* e caratteristiche ecoclimatiche delle località di campionamento in Africa Occidentale. Parassitologia, 32(S): 66-67.
203. DELLA TORRE A., MERZAGORA L., FORTINI D., COLUZZI M. (1990). Distribuzione in un apparato a due camere di scelta di *Anopheles gambiae* portatori di ordinamenti alternativi dell'inversione *2La*. Parassitologia, 32(S): 78-79.
204. DELLA TORRE A., MERZAGORA L., PETRANGELI G., COLUZZI M. (1990). Acquisizione di polimorfismi cromosomici attraverso introgressione tra specie gemelle del complesso *Anopheles gambiae*. Parassitologia, 32(S): 80-81.
205. IORI A., CANCRINI G., VEZZONI A., DEL NINNO G., TASSI P., GENCHI C., DELLA TORRE A., COLUZZI M. (1990). Osservazioni sul ruolo di *Culex pipiens* nella trasmissione della filariosi del cane in Italia. Parassitologia, 32(S): 151-152.
206. RALISOA O., SABATINI A., RAKOTOSON R., BOCCOLINI D., RANDRIA-NARISOA E., COLUZZI M. (1990). Rôle d'*Anopheles arabiensis* dans la transmission de *Plasmodium falciparum* dans la zone d'Antananarivo, Madagascar. Parassitologia, 32(S): 220-221.
207. SABATINI A., RAINERI V., TROVATO G., COLUZZI M. (1990). *Aedes albopictus* in Italia e possibile diffusione della specie nell'area mediterranea. Parassitologia, 32(S): 301-304.
208. ZHENG L., SAUNDERS R.D.C., FORTINI D., DELLA TORRE A., COLUZZI M., GLOVER D.M., KAFATOS F.C. (1991). Low-resolution genome map of the malaria mosquito *Anopheles gambiae*. Proceedings of the National Academy of Sciences of the USA, 88: 11187-11191.
209. CANCRINI G., D'AMELIO S., MATTIUCCI S., COLUZZI M. (1991). Identification of *Dirofilaria* in man by multilocus electrophoretic analysis. Annals of Tropical Medicine and Parasitology, 85: 529-532.
210. RAINERI V., TROVATO G., SABATINI M., COLUZZI M. (1991). Ulteriori dati sulla diffusione a Genova di *Aedes albopictus*. Parassitologia, 33: 183-185.
211. RALISOA O., RASAMINDRAKOTROKA A., RAVELOJANA B., RAFARASOA L., RANDRIANARISOA E., RAKOTOSON R., BOSMAN A., COLUZZI M. (1991). *Plasmodium falciparum* malaria transmission indices in a highland village of the Ikopa river valley near Antananarivo, Madagascar. Parassitologia, 33: 219-223.
212. COLUZZI M. (1992). Malaria vector analysis and control. Parasitology Today, 8: 113‑118.
213. BOSMAN A., COLUZZI M. (1992). Malaria urbana nella regione afrotropicale. L'Igiene moderna, 97: 797-806.
214. RALISOA O., BOCCOLINI D., RAKOTOSON R., SABATINI A., RANDRIANA-RISOA E., RAFARASOA L., COLUZZI M. (1992). Overwintering of *Anopheles funestus* and malaria control in the highlands of Madagascar. In: Proceedings of the VIth European Multicolloquium of Parasitology, The Hague, September 7-11, 1992, Abs. 15/16, p. 48.
215. CANCRINI G., DELLA TORRE A., COLUZZI M. (1992). Different probabilities of transmission of *Dirofilaria immitis* and *D. repens* by *Culex pipiens*. In: Proceedings of the VIth European Multicolloquium of Parasitology, The Hague, September 7-11, 1992, Abs. 116, p. 90.
216. DELLA TORRE A., SABATINI A., BOCCOLINI D., COLUZZI M. (1992). Variation in the palpal index of *Anopheles gambiae* and its association with the *2La* inversion polymorphism. In: Proceedings of the VIth European Multicolloquium of Parasitology, The Hague, September 7-11, 1992, Abs. 130, p. 130.
217. AKOGBETO M., MUKABAYIRE O., DI DECO M.A., ROMANO R., COLUZZI M. (1992). Different patterns of *Plasmodium falciparum* transmission in Northern Borgou, Benin, West Africa. In: Proceedings of the VIth European Multicolloquium of Parasitology, The Hague, September 7-11, 1992, Abs. P99, p. 162.
218. BOCCOLINI D., RAKOTOSON R., RALISOA O., SABATINI A., RANDRIA-NARISOA E., COLUZZI M. (1992). Polimorfismo cromosomico di *Anopheles funestus* in Madagascar. Parassitologia, 34(S): 14-15.
219. AWAHMUKALAH D.S.T., COLUZZI M., PETRARCA V. (1992). Osservazioni sul complesso *Anopheles gambiae* (Diptera, Culicidae) in Africa occidentale. Parassitologia, 34(S): 18-19.
220. AKOGBETO M., MUKABAYIRE O., DI DECO M.A., COLUZZI M. (1992). Osservazioni sulla distribuzione ecogeografica e stagionale dei membri del complesso *Anopheles gambiae* nel nord del Borgou, Benin, Africa occidentale. Parassitologia, 34(S): 20-21.
221. SABATINI A., RALISOA O., BOCCOLINI D., RANDRIANARISOA E., RAKOTOSON R., COLUZZI M. (1992). Differenze tra gli indici palpali di *Anopheles gambiae* portatori di cariotipi alternativi dell'inversione *2La*. Parassitologia, 34(S): 235-236.
222. FONTENILLE D., LEPERS J.P., COLUZZI M., CAMPBELL G.H., RAKOTO-ARIVONY I., COULANGES P. (1992). Malaria transmission and vector biology on Sainte Marie Island, Madagascar. Journal of Medical Entomology, 29: 197-202.
223. ROBERT V., VAN DE BROEK A., STEVENS P., SLOOTWEG R., PETRARCA V., COLUZZI M., LE GOFF G., DI DECO M.A., CARNEVALE P. (1992). Mosquitoes and malaria transmission in irrigated rice-fields in the Benoue valley of northern Cameroun. Acta Tropica, 52: 201-204.
224. COLUZZI M. (1993). Modificazioni ambientali e malaria nell'Africa subsahariana. Atti dei Convegni Lincei, 102: 97-106.
225. COLUZZI M. (1993). Advances in the study of Afrotropical malaria vectors. Parassitologia, 35: 23-29.
226. TAYLOR C.E., TOURÉ Y.T., COLUZZI M., PETRARCA V. (1993). Effective population size and persistence of *Anopheles arabiensis* during the dry season in West Africa. Medical and Veterinary Entomology, 7: 351-357.
227. COSTANTINI C., GIBSON G., BRADY J., MERZAGORA L., COLUZZI M. (1993). A new odour-baited trap to collect host-seeking mosquitoes. Parassitologia, 35: 5-9.
228. MILLIGAN P.J.M., PHILLIPS A., BROOMFIELD G., MOLYNEUX D.H., TOURÉ Y., COLUZZI M. (1993). A study of the use of gas chromatography of cuticular hydrocarbons for identifying members of the *Anopheles gambiae* (Diptera: Culicidae) complex. Bullettin of Entomological Research, 83: 613-624.
229. BOCCOLINI D., SABATINI A., SANOGO E., SAGNON N., COLUZZI M., COSTANTINI C. (1994). Chromosomal and vectorial heterogeneities in *Anopheles funestus* from Burkina Faso, West Africa. Parassitologia, 36(S): 20.
230. TOURÉ Y.T., PETRARCA V., TRAORÉ S.F., COULIBALY A., MAIGA H.M., SANKARÉ O., SOW M., DI DECO M.A., COLUZZI M. (1994). Ecological genetic studies in the chromosomal form Mopti of *Anopheles gambiae* s.str. in Mali, West Africa. Genetica, 94: 213-223.
231. COLUZZI M. (1994). Malaria and the Afrotropical ecosystems: impact of man-made environmental changes. Parassitologia, 36: 223-227.
232. FAVIA G., DIMOPOULOS G., DELLA TORRE A., TOURÉ Y.T., COLUZZI M., LOUIS C. (1994). Polymorphism detected by random PCR distinguish between different chromosomal forms of *Anopheles gambiae*. Proceedings of the National Academy of Sciences of the USA, 91: 10315-10319.
233. DELL'ANNA L., UTZERI C., SABATINI A., COLUZZI M. (1995). *Forcipomyia* (*Pterobosca*) *paludis* (Macfie, 1936) (Diptera, Ceratopogonidae) on adult dragonflies (Odonata) in Sardinia, Italy. Parassitologia, 37: 79-82.
234. COLUZZI M., CORBELLINI G. (1995). I luoghi della mal'aria e le cause della malaria. Medicina nei Secoli Arte e Scienza, 7: 575-598.
235. MODIANO D., PETRARCA V., SIRIMA B.S., BOSMAN A., NEBIÉ I., DIALLO D., LAMIZANA L., ESPOSITO F., COLUZZI M. (1995). *Plasmodium falciparum* malaria in sympatric ethnic groups of Burkina Faso (West Africa). Parassitologia, 37: 255-259.
236. TOURÉ Y.T., PETRARCA V., DI DECO M.A., TRAORÉ S.F., SAKAI S., COLUZZI M. (1996). Incipient species within *Anopheles gambiae* s.s. in West Africa. Parassitologia, 38: 10.
237. COSTANTINI C., LI S.G., DELLA TORRE A., SAGNON N'F., COLUZZI M. , TAYLOR C.E. (1996). Density, survival and dispersal of *Anopheles gambiae* complex mosquitoes in a West African Sudan savanna village. Medical and Veterinary Entomology, 10: 203-219.
238. COSTANTINI C., GIBSON G., SAGNON N'F., DELLA TORRE A., BRADY J., COLUZZI M. (1996). Mosquito responses to carbon dioxide in a West African Sudan savanna village. Medical and Veterinary Entomology, 10: 220-227.
239. DELLA TORRE A., FAVIA G., MARIOTTI G., COLUZZI M., MATHIOPOULOS K. (1996). Physical map of the malaria vector *Anopheles gambiae*. Genetics, 143: 1307-1311.
240. MODIANO D., PETRARCA V., SIRIMA B.S., NEBIÉ I., DIALLO D., ESPOSITO F., COLUZZI M. (1996). Different response to *Plasmodium falciparum* malaria in West African sympatric ethnic groups. Proceedings of the National Academy of Sciences of the USA, 93: 13206-13211.
241. FAVIA G., LANFRANCOTTI A., DELLA TORRE A., CANCRINI G., COLUZZI M. (1996). Polymerase chain reaction-identification of *Dirofilaria repens* and *Dirofilaria immitis*. Parasitology, 113: 567-571.
242. COLUZZI M. (1997). Interazioni evolutive uomo – plasmodio – anofele. In: Atti del XXIII Seminario sulla Evoluzione Biologica: "Coevoluzione e coadattamento". Accademia Nazionale dei Lincei , 22-24 febbraio 1996, pp. 263-285.
243. DELLA TORRE A., MERZAGORA L., POWELL J.R., COLUZZI M. (1997) Selective introgression of paracentric inversions between two sibling species of *Anopheles gambiae* complex. Genetics, 146: 239-244
244. FAVIA G., DELLA TORRE A., BAGAYOKO M., LANFRANCOTTI A., SAGNON N'F., TOURE' Y.T., COLUZZI M. (1997) Molecular identification of sympatric chromosomal forms of *Anopheles gambiae* and further evidence of their reproductive isolation. Insect Molecular Biology, 6: 377-383
245. FAVIA G., LANFRANCOTTI A., DELLA TORRE A., CANCRINI G., COLUZZI M. (1997). Advances in the identification of *Dirofilaria repens* and *Dirofilaria immitis* by a PCR-based approach. Parassitologia, 39: 401-402.
246. LOCHOUARN L., DIA I., BOCCOLINI D., COLUZZI M., FONTENILLE D. (1998). Bionomical and cytogenetic heterogeneities of *Anopheles funestus* in Senegal. Transactions of the Royal Society of Tropical Medicine and Hygiene, 92: 607-612.
247. COSTANTINI C., SAGNON N'F., DELLA TORRE A., DIALLO M., BRADY J., GIBSON G., COLUZZI M. (1998). Odor-mediated host preferences of East African mosquitoes, with particular reference to malaria vectors. American Journal of Tropical Medicine and Hygiene, 58: 56-63.
248. MODIANO D., CHIUCCHIUINI A., PETRARCA V., SIRIMA B.S., PERLMAN H., ESPOSITO F., COLUZZI M. (1998). Humoral response to *Plasmodium falciparum* pf155 and pf 332 in three sympatric ethnic groups of Burkina Faso. American Journal of Tropical Medicine and Hygiene, 58: 220-224.
249. MODIANO D., PETRARCA V., SIRIMA B.S., NEBIE I., LUONI G., ESPOSITO F., COLUZZI M .(1998). Baseline immunity of the population and impact of insecticide-treated curtains on malaria infection. American Journal of Tropical Medicine and Hygiene, 59: 336-340.
250. MATHIOPOULOS K.D., DELLA TORRE A., PREDAZZI V., PETRARCA V., COLUZZI M. (1998). Cloning inversions breakpoints in the *Anopheles gambiae* complex traces a transposable element at the inversion junction. Proceedings of the National Academy of Sciences of the USA, 95: 12444-12449.
251. COSTANTINI C., SAGNON N'F., SANOGO E., MERZAGORA L., COLUZZI M., (1998). Relationship to human bites collection and influence of light and bednet in CDC light-trap catches of West African malaria vectors. Bulletin of Entomological Research, 88: 503-511.
252. COLUZZI M., CORBELLINI G. (1998). Il centenario della malariologia (1898-1998). Parassitologia, 40: 361-375.
253. TOURÉ Y.T., PETRARCA V., TRAORÉ S.F., COUBALY A., MAIGA H.M., SANKARE’ O., SOW M., DI DECO M.A., COLUZZI M. (1998). The distribution and inversion polymorphism of chromosomally recognized taxa of the *Anopheles gambiae* complex in Mali, West Africa. Parassitologia, 40: 477-511.
254. ARCÀ B., LOMBARDO F., DE LARA CAPURRO M., DELLA TORRE A., DIMOPOULOS G., JAMES A.A., COLUZZI M. (1999). Trapping cDNAs encoding proteins from the salivary glands of the malaria mosquito *Anopheles gambiae*. Proceedings of the National Academy of Sciences of the USA, 96: 1516-1521.
255. Powell J.R., Petrarca V., della Torre A., Caccone A., Coluzzi M. (1999). Population structure, speciation, and introgression in the *Anopheles gambiae* complex. Parassitologia, 41: 101-113
256. MATHIOPOULOS K.D., DELLA TORRE A., SANTOLAMAZZA F., PREDAZZI V., PETRARCA V., COLUZZI M. (1999). Are chromosomal inversions induced by transposable elements? A paradigm from the malaria mosquito *Anopheles gambiae*. Parassitologia, 41: 119-123.
257. Modiano d., Petrarca V., Sirima b.s., luoni g., Nebie i., Diallo d.a., Esposito f., Coluzzi m. (1999). Different response to *Plasmodium falciparum* in West African sympatric ethnic groups: possible implications for malaria control strategies. Parassitologia, 41: 193-197.
258. COSTANTINI C., SAGNON N'F., DELLA TORRE A., COLUZZI M. (1999). Mosquito behavioural aspects of vector-human interactions in the *Anopheles gambiae* complex. Parassitologia, 41: 209-217.
259. COLUZZI M. (1999). The clay feet of the malaria giant and its African roots: hypotheses and inferences about origin, spread and control of *Plasmodium falciparum*. Parassitologia, 41: 277-283.
260. ARCÀ B., LOMBARDO F., DE LARA CAPURRO M., DELLA TORRE A., SPANOS L., DIMOPOULOS G., LOUIS C., JAMES A.A., COLUZZI M. (1999). Salivary gland-specific gene expression in the malaria vector *Anopheles gambiae*. Parassitologia, 41: 483‑487.
261. COSTANTINI C., SAGNON N'F., ILBOUDO-SANOGO E., COLUZZI M., BOCCOLINI D. (1999). Chromosomal and bionomic heterogeneities suggest incipient speciation in *Anopheles funestus* from Burkina Faso. Parassitologia, 41: 595-611.
262. MODIANO D., CHIUCCHIUINI A., PETRARCA V., SIRIMA B.S., LUONI G., ROGGERO M.A., CORRADIN G., COLUZZI M., ESPOSITO F. (1999). Interethnic differences in the humoral response to non-repetitive regions of the *Plasmodium falciparum* circumsporozoite protein. American Journal of Tropical Medicine and Hygiene, 61: 663‑667.
263. COLUZZI M. (2000). Eradicazione della malaria in Calabria, anofelismo residuo e rischio di trasmissione. Parassitologia, 42: 211-217.
264. ARCÀ B., LOMBARDO F., LANFRANCOTTI A., SPANOS L., VENERI M., LOUIS C., COLUZZI M. (2000). A cluster of D7‑related genes is expressed in the salivary glands of the African malaria vector *Anopheles gambiae*. Parassitologia, 42(S): 123.
265. COSTANTINI C., BIRKETT M., SAGNON N'F., COLUZZI M., PICKETT J. (2000). Two human‑specific acids reduce the field trap response to carbon dioxide of *Anopheles gambiae* s.l. Parassitologia, 42(S): 128.
266. DELLA TORRE A., FANELLO C., AKOGBETO M., DOSSOU‑YOVO J., FAVIA G., PETRARCA V., COLUZZI M. (2000). PCR‑RFLP analysis reveals further complexities within *Anopheles gambiae* s.s. in West Africa. Parassitologia, 42(S): 130.
267. DOLO A., MODIANO D., GUINDO H., DOLO G., MAIGA B., COULIBALY D., TOURÉ Y., COLUZZI M., DOUMBO O. (2000). Interethnic comparisons of malaria susceptibility in Mali. Parassitologia, 42(S): 131.
268. FANELLO C., DELLA TORRE A., DOSSOU‑YOVO J., PETRARCA V., CURTIS C., COLUZZI M. (2000). The *kdr pyrethroid resistance* gene as a marker of gene flow among wild populations of the malaria vector *Anopheles gambiae*. Parassitologia, 42(S): 132.
269. LANFRANCOTTI A., LOMBARDO F., COLUZZI M., ARCÀ B. (2000). Identification of novel salivary gland genes from the malaria mosquito *Anopheles gambiae* by the Signal Sequence Trap. Parassitologia, 42(S): 135.
270. LOMBARDO F., DI CRISTINA M., SPANOS L., LOUIS C., COLUZZI M., ARCÀ B. (2000). The *Anopheles gambiae* salivary *apyrase*: promoter sequences of the mosquito gene confer salivary gland expression in *Drosophila melanogaster*. Parassitologia, 42(S): 136.
271. LOMBARDO F., DI CRISTINA M., SPANOS L., LOUIS C., COLUZZI M., ARCÀ B. (2000). Promoter sequences of the putative *Anopheles gambiae apyrase* confer salivary gland expression in *Drosophila melanogaster*. Journal of Biological Chemistry, 275: 23861-23868.
272. LUONI G., ARCÀ B., SIRIMA B.S., VERRA F., COLUZZI M., MODIANO D. (2000). Analysis of the IL‑4 promoter region in three West African ethnic groups characterized by different immune reactivity to *Plasmodium falciparum* malaria. Parassitologia, 42(S): 137.
273. MODIANO D., LUONI G., SIRIMA B.S., LANFRANCOTTI A., PETRARCA V., CRUCIANI F., SIMPORÉ J., CIMINELLI B.M., FOGLIETTA E., GRISANTI P., BIANCO I., MODIANO G., COLUZZI M. (2000). The lower susceptibility to *Plasmodium falciparum* malaria in West African Fulani compared to sympatric populations is observed in spite of lower frequencies of classical malaria resistance alleles. Parassitologia, 42(S): 139.
274. SAGNON N'F., COSTANTINI C., DELLA TORRE A., COLUZZI M. (2000). Non‑random distribution of larval populations of sympatric members of the *Anopheles gambiae* complex in Burkina Faso. Parassitologia, 42(S): 157.
275. PETRARCA V., NUGUD A.D., ELKARIM AHMED M.A., HARIDI A.M., DI DECO M.A., COLUZZI M. (2000). Cytogenetics of the *Anopheles gambiae* complex in Sudan, with special reference to *An. arabiensis*: relationships with East and West African populations. Medical and Veterinary Entomology, 14: 149-164.
276. TOURÉ Y.T., COLUZZI M. (2000). The challenges of doing more against malaria, particularly in Africa. Bulletin of the World Health Organization, 78: 1376.
277. COLUZZI M. (2000). Ipotesi ed inferenze sull'origine e la diffusione di *Plasmodium falciparum* e strategie di controllo. Giornale dell’Accademia di Medicina di Torino, CLXIII: 213-225.
278. ARCÀ B., LOMBARDO F., LANFRANCOTTI A., COLUZZI M. (2000). Malaria: prospettive biotecnologiche di lotta al vettore. Giornale dell’Accademia di Medicina di Torino, CLXIII: 226-239.
279. MODIANO D., COLUZZI M. (2000). Le malarie da *Plasmodium falciparum*. Giornale dell’Accademia di Medicina di Torino, CLXIII: 240-253.
280. DUCHEMIN J.B., LEONG POCK TSY J.M., RABARISON P., ROUX J., COLUZZI M., COSTANTINI C. (2001). Zoophily of *Anopheles arabiensis* and *An. gambiae* in Madagascar demonstrated by odour-baited entry traps. Medical and Veterinary Entomology 15: 50‑57.
281. DELLA TORRE A., FANELLO C., AKOGBETO M., DOSSOU-YOVO J., FAVIA G., PETRARCA V., COLUZZI M. (2001). Molecular evidence of incipient speciation within *Anopheles gambiae* s.s. in West Africa. Insect Molecular Biology, 10: 9-18.
282. COSTANTINI C., BIRKETT M.A., GIBSON G., ZIESMANN J., SAGNON N'F., MOHAMMED H.A., COLUZZI M., PICKETT J.A. (2001). Electroantennogram and behavioural responses of the malaria vector *Anopheles gambiae* to human-specific sweat components. Medical and Veterinary Entomology, 15: 259-266.
283. LUONI G., VERRA F., ARCÀ B., SIRIMA B.S., TROYE-BLOMBERG M., COLUZZI M., KWIATKOWSKI D., MODIANO D. (2001). Antimalarial antibody levels and *IL4* polymorphism in the Fulani of West Africa. Genes and Immunity, 2: 411-414.
284. MODIANO D., LUONI G., PETRARCA V., SIRIMA B.S., DE LUCA M., SIMPORÉ J., COLUZZI M., BODMER J.G., MODIANO G. (2001). HLA class I in three West African ethnic groups: genetic distances from sub-Saharan and Caucasoid populations. Tissue Antigens, 57: 128-137.
285. MODIANO D., LUONI G., SIRIMA B.S., LANFRANCOTTI A., PETRARCA V., CRUCIANI F., SIMPORE J., CIMINELLI B.M., FOGLIETTA E., GRISANTI P., BIANCO I., MODIANO G., COLUZZI M. (2001). The lower susceptibility to *Plasmodium falciparum* malaria of Fulani of Burkina Faso (West Africa) is associated with low frequencies of classic malaria-resistance genes. Transactions of the Royal Society of Tropical Medicine and Hygiene, 95: 149-152.
286. MODIANO D., LUONI G., SIRIMA B.S., SIMPORÉ J., VERRA F., KONATÉ A., RASTRELLI E., OLIVIERI A., CALISSANO C., PAGANOTTI G.M., D'URBANO L., SANOU I., SAWADOGO A., MODIANO G., COLUZZI M. (2001). Haemoglobin C protects against clinical *Plasmodium falciparum* malaria. Nature, 414: 305-308.
287. MUKABAYIRE O., CARIDI J., WANG X., TOURÉ Y.T., COLUZZI M., BESANSKY N.J. (2001). Patterns of DNA sequence variation in chromosomally recognized taxa of *Anopheles gambiae*: evidence from rDNA and single-copy loci. Insect Molecular Biology, 10: 33-46.
288. ARCÀ B., LOMBARDO F., LANFRANCOTTI A., SPANOS L., VENERI M., LOUIS C., COLUZZI M. (2002). A cluster of four *D7-related* genes is expressed in the salivary glands of the African malaria vector *Anopheles gambiae*. Insect Molecular Biology, 11: 47‑55.
289. COLUZZI M. (2002). Ettore Biocca, un irraggiugibile esempio di vita. Parassitologia, 44: 1‑4.
290. COLUZZI M., MANNO D., GUZZINATI S., TOGNAZZO S., ZAMBON P., ARCÀ B., COSTANTINI C., ASCOLI V. (2002). The bloodsucking arthropod bite as possible cofactor in the transmission of human herpesvirus-8 infection and in the expression of Kaposi's sarcoma disease. Parassitologia, 44: 123-129.
291. COLUZZI M. (2002). *Plasmodium falciparum* en Afrique subsaharienne - Spéciation récente des vecteurs, transmissibilité, évolution de la pathogenèse / controle de la maladie, et capacité vectorielle. Annales de l'Institut Pasteur/actualités, 13: 81-99.
292. DELLA TORRE A., COSTANTINI C., BESANSKY N.J., CACCONE A., PETRARCA V., POWELL J.R., COLUZZI M. (2002). Speciation within *Anopheles gambiae* - the glass is half full. Science, 298: 115-117.
293. HOLT R.A. *et al*. (2002). The genome sequence of the malaria mosquito *Anopheles gambiae*. Science, 298: 129-149.
294. COLUZZI M., SABATINI A., DELLA TORRE A., DI DECO A., PETRARCA V. (2002). A polytene chromosome analysis of the *Anopheles gambiae* complex. Science, 298: 1415-1418.
295. LANFRANCOTTI A., LOMBARDO F., SANTOLAMAZZA F., VENERI M., CASTRIGNANÒ T., COLUZZI M., ARCÀ B. (2002). Novel cDNAs encoding salivary proteins from the malaria vector *Anopheles gambiae*. FEBS Letters, 517: 67-71.
296. ASCOLI V., MANNO D., GUZZINATI S., TOGNAZZO S., ZAMBON P., ARCA' B., COSTANTINI C., COLUZZI M. (2002). La puntura di artropodi ematofagi quale possibile cofattore nella trasmissione di HHV8 (Human Herpes Virus 8) e nell'espressione del sarcoma di Kaposi. Accademia Nazionale dei Lincei, Rendiconti della Classe di Scienze fisiche, matematiche e naturali, 13: 71-88.
297. COLUZZI M., COSTANTINI C. (2002). An alternative focus in strategic research on disease vectors: the potential of genetically modified non‑biting mosquitoes. Parassitologia 44: 131-135.
298. COLUZZI M. (2002). Stato palustre ed anofelico (Paludismo) senza malaria – Editorial introduction to the paper by A. Celli and G. Gasperini reprinted from the Atti della Società per gli studii della Malaria (Volume III, 1902, pp. 115-145). Parassitologia, 44: 207-212.
299. ASCOLI V., ZAMBON P., MANNO D., GUZZINATI S., ZORZI M., ARCA’ B., COSTANTINI C., COLUZZI M. (2003). Variability in the incidence of classic Kaposi's sarcoma in the Veneto region, Northern Italy. Tumori, 89: 122-124.
300. FANELLO C., PETRARCA V., DELLA TORRE A., SANTOLAMAZZA F., DOLO G., COULIBALY M., ALLOUECHE A., CURTIS C.F., TOURE’ Y.T., COLUZZI M. (2003). The pyrethroid *knock-down resistance* gene in the *Anopheles gambiae* complex in Mali and further indication of incipient speciation within *An. gambiae* s.s. Insect Molecular Biology 12: 241-245.
301. PAGANOTTI G.M., PALLADINO C., COLUZZI M. (2003). L'evoluzione della malaria. Le Scienze, 420: 70-78.
302. COLUZZI M., CALABRO’ M., MANNO D., CHIECO-BIANCHI L., SCHULZ T.F., ASCOLI V. (2003). Reduced seroprevalence of Kaposi’s sarcoma-associated herpesvirus (KSHV), human herpesvirus 8 (HHV8), related to suppression of *Anopheles* density in Italy. Medical and Veterinary Entomology, 17: 461-464.
303. PAGANOTTI G.M., PALLADINO C., COLUZZI M. (2004). Der Ursprung der Malaria. Spektrum der Wissenschaft, Marz: 82-89.

**304.** COLUZZI M, RIOUX JA. (2004). [The 2003 International E. Brumpt Prize]. Bull Soc Pathol Exot. 97: 371-374.

**305**. VERRA F, LUONI G, CALISSANO C, TROYE-BLOMBERG M, PERLMANN P, PERLMANN H, ARCÀ B, SIRIMA BS, KONATÉ A, COLUZZI M, KWIATKOWSKI D, MODIANO D. (2004). IL4-589C/T polymorphism and IgE levels in severe malaria. Acta Trop. 90: 205-209.

**306.** COLUZZI M, CALABRÒ ML, MANNO D, CHIECO-BIANCHI L, SCHULZ TF, ASCOLI V. (2004). Saliva and the transmission of human herpesvirus 8: potential role of promoter-arthropod bites. J Infect Dis. 190: 199-200; author reply 200-201.

**307.** PAGANOTTI GM, BABIKER HA, MODIANO D, SIRIMA BS, VERRA F, KONATÉ A, OUEDRAOGO AL, DIARRA A, MACKINNON MJ, COLUZZI M, WALLIKER D. (2004) Genetic complexity of *Plasmodium falciparum* in two ethnic groups of Burkina Faso with marked differences in susceptibility to malaria. Am J Trop Med Hyg. 71: 173-178.

**308.** COLUZZI M, CALABRÒ ML, MANNO D, CHIECO-BIANCHI L, SCHULZ TF, ASCOLI V. (2004). HHV-8 transmission via saliva to soothe blood-sucking arthropod bites. Br J Cancer. 91: 998-999; author reply 999.

**309.** CELLI A, COLUZZI M. (2004). [Italian state's quinine laws: the role of Giustino Fortunato and Angelo Celli. An introduction to the paper by Anna Celli "La lotta contro la malaria" reprinted form "Giustino Fortunato (1848-1932)," Ed. Archivio Storico per la Calabria e la Lucania, 1932. pp. 135-153]. Parassitologia 46: 329-347; discussion 327-328.

**310.** SIMPORE J, GRANATO M, SANTARELLI R, NSME RA, COLUZZI M, PIETRA V, PIGNATELLI S, BERE A, FAGGIONI A, ANGELONI A. (2004). Prevalence of infection by HHV-8, HIV, HCV and HBV among pregnant women in Burkina Faso. J Clin Virol. 31: 78-80.

**311.** AMOROSA LF JR, CORBELLINI G, COLUZZI M. (2005). Lessons learned from malaria: Italy's past and sub-Sahara's future. Health Place. 11: 67-73.

**312.** DOLO A, MODIANO D, MAIGA B, DAOU M, DOLO G, GUINDO H, BA M, MAIGA H, COULIBALY D, PERLMAN H, BLOMBERG MT, TOURÉ YT, COLUZZI M, DOUMBO O. (2005). Difference in susceptibility to malaria between two sympatric ethnic groups in Mali. Am J Trop Med Hyg. 72: 243-248.

**313.** LOMBARDO F, NOLAN T, LYCETT G, LANFRANCOTTI A, STICH N, CATTERUCCIA F, LOUIS C, COLUZZI M, ARCÀ B. (2005). An *Anopheles gambiae* salivary gland promoter analysis in *Drosophila melanogaster* and *Anopheles stephensi*. Insect Mol Biol. 14: 207-216.

**314.** AYALA FJ, COLUZZI M. (2005). Chromosome speciation: humans, *Drosophila*, and mosquitoes. Proc Natl Acad Sci U S A. 102 Suppl 1: 6535-6542. Epub 2005 Apr 25.

**315.** ARCÀ B, LOMBARDO F, VALENZUELA JG, FRANCISCHETTI IM, MARINOTTI O, COLUZZI M, RIBEIRO JM. (2005). An updated catalogue of salivary gland transcripts in the adult female mosquito, *Anopheles gambiae*. J Exp Biol. 208: 3971-3986.

**316.** CAPUTO B, DANI FR, HORNE GL, PETRARCA V, TURILLAZZI S, COLUZZI M, PRIESTMAN AA, DELLA TORRE A. (2005). Identification and composition of cuticular hydrocarbons of the major Afrotropical malaria vector *Anopheles gambiae* s.s. (Diptera: Culicidae): analysis of sexual dimorphism and age-related changes. J Mass Spectrom. 40: 1595-1604.

**317.** PAGANOTTI GM, PALLADINO C, MODIANO D, SIRIMA BS, RÅBERG L, DIARRA A, KONATÉ A, COLUZZI M, WALLIKER D, BABIKER HA. (2006). Genetic complexity and gametocyte production of *Plasmodium falciparum* in Fulani and Mossi communities in Burkina Faso. Parasitology. 132: 607-614. Epub 2006 Jan 18.

**318.** ASCOLI V, FACCHINELLI L, VALERIO L, ZUCCHETTO A, DAL MASO L, COLUZZI M. (2006). Distribution of mosquito species in areas with high and low incidence of classic Kaposi's sarcoma and seroprevalence for HHV-8. Med Vet Entomol. 20: 198-208.

**319.** CHIRONNA M, TOSATTI MA, DI GANGI IM, SALLUSTIO A, GERMINARIO C, COLUZZI M, QUARTO M, CHIECO-BIANCHI L, CALABRÒ ML. (2006). High human herpesvirus 8 seroprevalence in populations from Western Balkan countries. J Med Virol. 78: 933-937.

**320.** ASCOLI V, MANNO D, COLUZZI M. Geographic variation in human herpesvirus 8 seroprevalence and possible association with exposure to bites from blood-sucking arthropods. J Infect Dis. 194: 401-402; author reply 402.

**321.** ASCOLI V, FACCHINELLI L, VALERIO L, MANNO D, COLUZZI M. (2006). Kaposi's sarcoma, human herpesvirus 8 infection and the potential role of promoter-arthropod bites in northern Sweden. J Med Virol. 78: 1452-1455.

**322.** LOMBARDO F, LANFRANCOTTI A, MESTRES-SIMÓN M, RIZZO C, COLUZZI M, ARCÀ B. (2006). At the interface between parasite and host: the salivary glands of the African malaria vector *Anopheles gambiae*. Parassitologia. 48: 573-580.

**323.** BIETOLINI S, CANDURA F, COLUZZI M. (2006). Spatial and long term temporal distribution of the *Anopheles maculipennis* complex species in Italy. Parassitologia. 48: 581-608.

**324.** CAPUTO B, DANI FR, HORNE GL, N'FALE S, DIABATE A, TURILLAZZI S, COLUZZI M, COSTANTINI C, PRIESTMAN AA, PETRARCA V, DELLA TORRE A. (2007). Comparative analysis of epicuticular lipid profiles of sympatric and allopatric field populations of *Anopheles gambiae* s.s. molecular forms and *An. arabiensis* from Burkina Faso (West Africa). Insect Biochem Mol Biol. 37: 389-398.

**325.** TORCIA MG, SANTARLASCI V, COSMI L, CLEMENTE A, MAGGI L, MANGANO VD, VERRA F, BANCONE G, NEBIE I, SIRIMA BS, LIOTTA F, FROSALI F, ANGELI R, SEVERINI C, SANNELLA AR, BONINI P, LUCIBELLO M, MAGGI E, GARACI E, COLUZZI M, COZZOLINO F, ANNUNZIATO F, ROMAGNANI S, MODIANO D. (2008). Functional deficit of T regulatory cells in Fulani, an ethnic group with low susceptibility to *Plasmodium falciparum* malaria. Proc Natl Acad Sci U S A. 105: 646-651.

**326.** DELLA TORRE A, ARCA B, FAVIA G, PETRARCA V, COLUZZI M. (2008). The role of research in molecular entomology in the fight against malaria vectors. Parassitologia. 50: 137-140.

**327.** ESPOSITO F, MAJORI G, COLUZZI M. (2008). The role of research in the fight against malaria: the Italian contribution to malaria research in the frame of north-south cooperation in the last 25 years. Parassitologia. 50:141.

**328.** DANI FR, FRANCESE S, MASTROBUONI G, FELICIOLI A, CAPUTO B, SIMARD F, PIERACCINI G, MONETI G, COLUZZI M, DELLA TORRE A, TURILLAZZI S. (2008). Exploring proteins in *Anopheles gambiae* male and female antennae through MALDI mass spectrometry profiling. PLoS One. 3(7): e2822.

**329.** POMBI M, CAPUTO B, SIMARD F, DI DECO MA, COLUZZI M, DELLA TORRE A, COSTANTINI C, BESANSKY NJ, PETRARCA V. (2008). Chromosomal plasticity and evolutionary potential in the malaria vector *Anopheles gambiae* sensu stricto: insights from three decades of rare paracentric inversions. BMC Evol Biol. 8: 309.

**330.** COLUZZI M, GACHELIN G, HARDY A, OPINEL A. (2008). Insects and illnesses: contributions to the history of medical entomology. Introduction. Parassitologia. 50: 157-163.

**331.** LOMBARDO F, LYCETT GJ, LANFRANCOTTI A, COLUZZI M, ARCÀ B. (2009). Analysis of apyrase 5' upstream region validates improved *Anopheles gambiae* transformation technique. BMC Res Notes.2: 24.

**332.** LOMBARDO F, RONCA R, RIZZO C, MESTRES-SIMÒN M, LANFRANCOTTI A, CURRÀ C, FIORENTINO G, BOURGOUIN C, RIBEIRO JM, PETRARCA V, PONZI M, COLUZZI M, ARCÀ B. (2009). The *Anopheles gambiae* salivary protein gSG6: an anopheline-specific protein with a blood-feeding role. Insect Biochem Mol Biol. 39: 457-466.

**333.** ASCOLI V, SENIS G, ZUCCHETTO A, VALERIO L, FACCHINELLI L, BUDRONI M, DAL MASO L, COLUZZI M. (2009). Distribution of 'promoter' sandflies associated with incidence of classic Kaposi's sarcoma. Med Vet Entomol. 23: 217-225.

**334.** ROMANO R, TABACCHI F, PAGANOTTI GM, RUSSO G, GRAMOLELLI S, MARINUCCI F, CECCHERINI-NELLI L, COLUZZI M. (2010). Evaluation of bloodsucking arthropod bite as possible risk co-factor in Human herpesvirus-8 transmission route. Parassitologia. 52: 405-410.

**335.** RIZZO C, RONCA R, FIORENTINO G, VERRA F, MANGANO V, POINSIGNON A, SIRIMA SB, NÈBIÈ I, LOMBARDO F, REMOUE F, COLUZZI M, PETRARCA V, MODIANO D, ARCÀ B. (2011). Humoral response to the *Anopheles gambiae* salivary protein gSG6: a serological indicator of exposure to Afrotropical malaria vectors. PLoS One. 6(3): e17980.

**336.** DELLA TORRE A, POMBI M, PETRARCA V, COLUZZI M. (2011). New mosquito subgroup breeds questions. Science. 332 (6028): 419-20; author reply 420-1.

**337.** PAGANOTTI GM, GALLO BC, VERRA F, SIRIMA BS, NEBIÉ I, DIARRA A, COLUZZI M, MODIANO D. (2011). Human genetic variation is associated with *Plasmodium falciparum* drug resistance. J Infect Dis. 204: 1772-1778.

**338.** DRAGO A, MARINI F, CAPUTO B, COLUZZI M, DELLA TORRE A, POMBI M. (2012). Looking for the gold standard: assessment of the effectiveness of four traps for monitoring mosquitoes in Italy. J Vector Ecol. 37: 117-123.

**339.** PAGANOTTI GM, GRAMOLELLI S, TABACCHI F, RUSSO G, MODIANO D, COLUZZI M, ROMANO R. (2012). Distribution of human CYP2C8*2 allele in three different African populations. Malar J. 11: 125.

**BOOK CHAPTERS**

**B1** DAVIDSON G., PATERSON H.E., COLUZZI M., MASON G.F., MICKS D.W. (1967). The *Anopheles gambiae* complex. In: Genetics of Insects Vectors of Disease (Wright J.M., Pal R., eds) Elsevier Publ., Co. Amsterdam, 6: 211-250.

**B2** COLUZZI M., KITZMILLER J.B. (1975). Anopheline mosquitoes. In: Handbook of Genetics, vol 3 (King R.C., ed) Plenum Publishing Corp., New York, 3: 285-309.

**B3** BULLINI L., COLUZZI M. (1982). Evolutionary and taxonomic inferences of electrophoretic studies in mosquitoes. In: Recent Developments in the Genetics of Insect Disease Vectors, Stipes Publishing Co., Illinois, USA. pp. 465-482.

**B4** BRYAN J.H., DI DECO M.A., PETRARCA V., COLUZZI M. (1982). Further complexity in the *Anopheles gambiae* complex. In: Recent Developments in the Genetics of Insect Disease Vectors, Stipes Publishing Co., Illinois, USA. pp. 483-505.

**B5** COLUZZI M. (1982). Spatial distribution of chromosomal inversions and speciation in Anopheline mosquitoes. Mechanisms of Speciation, Liss, New York. pp. 143-153.

**B6** BRYAN J.H., PETRARCA V., DI DECO M.A., COLUZZI M. (1984). Mosquito behaviour studies in The Gambia, West Africa. In: Proceedings of a conference to honour Robert H. Black (Bryan J.H., Moodie P.M., eds) Australian Government Publishing Service, Canberra. pp. 157-160.

**B7** COLUZZI M. (1988). Anopheline mosquitos: genetic methods for species differentiation. In: Malaria Principles and Practice of Malariology (Wernsdorfer W.H., McGregor J., eds), Churchill Livingstone, Londra. pp. 411-428.

**B8** CURTIS C.F., LINES J.D., CARNEVALE P., ROBERT V., BOUDIN C., HALNA J-M., PAZART L., GAZIN P., RICHARD A., MOUCHET J., CHARLWOOD J.D., GRAVES P.M., HOSSAIN M.I., KURIHARA T., ICHIMORI K., LI ZUZI, LU BAOLIN, MAJORI G., SABATINELLI G., COLUZZI M., NJUNWA K.J., WILKES T.J., SNOW R.W., LINDSAY S.W. (1990). Impregnated bed nets and curtains against malaria mosquitoes. In: Appropriate Technology in Vector Control (Curtis C.F., ed). CRC Press Inc. Boca Raton, Florida, 2: 5-46.

**B9** COLUZZI M., SABATINI A. (1990). Anofeli. In: Atlante Tematico d'Italia. Touring Club Italiano, CNR. Vol. 30B.

**B10** CARPENTER C.C.J., BEIER J.C., CATTANI J.A., CHULAY J.D., COLUZZI M., COPPEL R.L., DOBERSTYN E.B., GEARY T.G., HAMMER J.S., HOFFMAN S.L., JENSEN J.B., KENDALL C., MILHOUS W.K., PIMENTEL D, TAUIL P.L., TAYLOR T.E., TEKLEHAIMANOT A., WARREN M., WOODWARD T.F. (1991). Malaria: Obstacles and Opportunities (Oaks S.C. Jr, Mitchell V.S., Pearson G.W., Carpenter C.C.J., eds) National Academy Press, Washington D.C. pp. 1-277.

**B11** COLUZZI M., SABATINI A. (1995). Diptera Culicomorpha, Culicidae. In: Checklist delle specie della fauna italiana (Generi 007-024) (Minelli A., Ruffo S., La Posta S., eds) Edizioni Calderini Bologna. pp. 65.9-65.11.
